# Solution epitaxy of polarization-gradient ferroelectric oxide films with colossal photovoltaic current

**DOI:** 10.1038/s41467-023-37823-z

**Published:** 2023-04-24

**Authors:** Chen Lin, Zijun Zhang, Zhenbang Dai, Mengjiao Wu, Shi Liu, Jialu Chen, Chenqiang Hua, Yunhao Lu, Fei Zhang, Hongbo Lou, Hongliang Dong, Qiaoshi Zeng, Jing Ma, Xiaodong Pi, Dikui Zhou, Yongjun Wu, He Tian, Andrew M. Rappe, Zhaohui Ren, Gaorong Han

**Affiliations:** 1 State Key Laboratory of Silicon and Advanced Semiconductor Materials, School of Materials Science and Engineering, Zhejiang University, Hangzhou, 310027 China; 2 Center of Electron Microscope, School of Materials Science and Engineering, Zhejiang University, Hangzhou, 310027 China; 3 Department of Chemistry, University of Pennsylvania, Philadelphia, PA 19104-6323 USA; 4 Oden Institute for Computational Engineering and Sciences, The University of Texas at Austin, Austin, Texas 78712 USA; 5 Key Laboratory for Quantum Materials of Zhejiang Province, Department of Physics, School of Science, Westlake University, Hangzhou, 310024 China; 6 Zhejiang Province Key Laboratory of Quantum Technology and Device, Department of physics, Zhejiang University, Hangzhou, 310027 China; 7 Center for High Pressure Science and Technology Advanced Research, Shanghai, 201203 China; 8 State Key Lab of New Ceramics and Fine Processing, School of Materials Science and Engineering, Tsinghua University, Beijing, 100091 China; 9 Institute of Advanced Semiconductors & Zhejiang Provincial Key Laboratory of Power Semiconductor Materials and Devices, ZJU-Hangzhou Global Scientific and Technological Innovation Center, Zhejiang University, Hangzhou, 311215 China; 10 Research Center for Intelligent Sensing, Zhejiang Lab, Hangzhou, 311100 China

**Keywords:** Ferroelectrics and multiferroics, Synthesis and processing

## Abstract

Solution growth of single-crystal ferroelectric oxide films has long been pursued for the low-cost development of high-performance electronic and optoelectronic devices. However, the established principles of vapor-phase epitaxy cannot be directly applied to solution epitaxy, as the interactions between the substrates and the grown materials in solution are quite different. Here, we report the successful epitaxy of single-domain ferroelectric oxide films on Nb-doped SrTiO_3_ single-crystal substrates by solution reaction at a low temperature of ~200 ^o^C. The epitaxy is mainly driven by an electronic polarization screening effect at the interface between the substrates and the as-grown ferroelectric oxide films, which is realized by the electrons from the doped substrates. Atomic-level characterization reveals a nontrivial polarization gradient throughout the films in a long range up to ~500 nm because of a possible structural transition from the monoclinic phase to the tetragonal phase. This polarization gradient generates an extremely high photovoltaic short-circuit current density of ~2.153 mA/cm^2^ and open-circuit voltage of ~1.15 V under 375 nm light illumination with power intensity of 500 mW/cm^2^, corresponding to the highest photoresponsivity of ~4.306×10^−3 ^A/W among all known ferroelectrics. Our results establish a general low-temperature solution route to produce single-crystal gradient films of ferroelectric oxides and thus open the avenue for their broad applications in self-powered photo-detectors, photovoltaic and optoelectronic devices.

## Abstract (web summary)

Developing low-temperature solution epitaxy and elucidating its underlying mechanisms is highly desired for low-cost fabrication of single-crystal ferroelectric films. Here, the authors show that a polarization screening between ferroelectrics and substrates can tailor the interface energy and drive the solution epitaxy of polarization-gradient ferroelectric oxide films, demonstrating a remarkable photovoltaic effect.

## Introduction

Ferroelectric oxide thin films, characterized by a switchable electric polarization, have been the focus of numerous investigations because of their intriguing properties and rich interfacial phenomena, as well as versatile potential applications^1–3^. Vapor-phase epitaxy has become well-established and widely adopted for designing and controlling the epitaxy of ferroelectric oxide films^4–6^ for discovering a wealth of fascinating effects, such as topological ferroelectricity^7–9^, improper ferroelectricity^10^ and negative capacitance^11^. Nevertheless, a high temperature (>600 ^o^C) is a prerequisite to obtain single-crystal films, leading to disparate elemental volatilities and interdiffusion of components near the interfaces^12–14^, which is not beneficial for device fabrication.

In the past decades, the low-temperature solution routes (100–200 ^o^C), including chemical solution deposition and hydrothermal epitaxy, have been intensively explored as a promising and economical approach to prepare ferroelectric oxide films. By these routes, the elemental volatility and interdiffusion of components during the film growth could be avoided in principle^4^. Polycrystalline films, however, were usually obtained by using a chemical solution deposition after a spin-coating process^15^. In the case of the hydrothermal epitaxy, the growth of ferroelectric oxide films is still largely empirical. In vapor-phase epitaxial growth, a single-crystal film can be formed via layer-by-layer growth (Frank-Van der Merwe growth). Such growth can be achieved thermodynamically by the wetting condition:

1 $${\sigma }_{{{{{{\rm{interface}}}}}}}+{\sigma }_{{{{{{\rm{surface}}}}}}} < {\sigma }_{{{{{{\rm{substrate}}}}}}}$$

The sum of the interface energy *σ*_interface_ and the surface energy *σ*_surface_ of the film is lower than the surface energy of the substrate *σ*_substrate_. Herein, one should note that *σ*_interface_ is dominated by the lattice mismatch, which is therefore decisive for the layer-by-layer epitaxial growth^8,16–18^. Unfortunately, this thermodynamic consideration in terms of the lattice mismatch alone does not guarantee a successful low-temperature solution epitaxy. For example, despite a very small lattice mismatch (≈0.03%) between PbTiO_3_ (PTO) and SrTiO_3_ (STO), continuous and smooth PTO film has not been obtained by a hydrothermal method, where PTO adopts an island growth (Volmer–Weber growth) mode^4,19^. Until now, it is still a formidable challenge to control the low-temperature solution epitaxy of ferroelectric films, because the growth mechanism has not been elucidated and the principles guiding their epitaxy remain exclusive.

In ferroelectric oxides, the presence of spontaneous polarization below the Curie temperature (*T*_c_) usually generates a depolarization field via surface-bound charges. This would make the polarization unstable, and thus screening or compensating these surface-bound charges is necessary for reducing the depolarization energy and stabilizing the polarization^20^. This can be achieved by nonstoichiometric surface reconstruction^21,22^, surface charge adsorption^23^, electrons or oxygen vacancies^24–28^. Although screening is a fundamental issue for ferroelectrics, its role in the epitaxial growth of ferroelectric films has not been sufficiently considered, particularly at low temperatures.

**Fig. 1:**
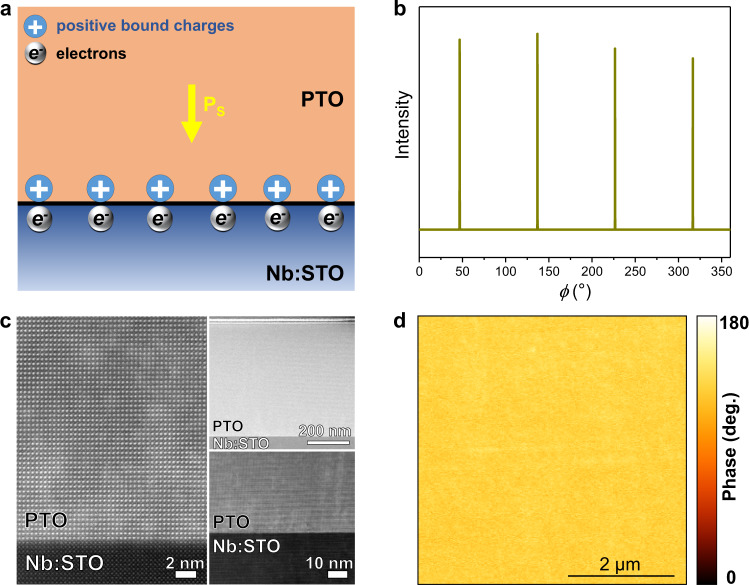
Electronic polarization screening driven solution epitaxy of single-crystal and single-domain PTO film on Nb:STO substrate. **a** Schematic illustration of the electronic polarization screening of PTO on Nb:STO substrate to lower the interface energy *σ*_interface_. **P**_**S**_ denotes the spontaneous polarization of PTO. **b** *ϕ* scan spectrum of PTO/Nb:STO along the pseudocubic <110> direction. **c** Cross-sectional HAADF-STEM images of PTO/Nb:STO with different magnifications. **d** Out-of-plane PFM phase image of PTO film.

Here, we propose that an electronic polarization screening at the interface between ferroelectric films and substrates is crucial for altering the interface energy (*σ*_interface_), allowing to control the growth mode and thus enable low-temperature solution epitaxy of single-crystal ferroelectric oxide films. To explore this proposition, PTO/STO system with a desirable lattice mismatch was chosen as a computational model to investigate the impact of doped substrates on the screening and the resulting film structure. The interface formation energy of PTO/Nb:STO (an *n*-type doped STO with Nb doping concentration of 10% was implemented due to the computational complexity) and PTO/STO systems have been investigated by first-principles calculations (Supplementary Fig. 1). Interestingly, the polarization of PTO, fully screened by charge (electrons) in Nb:STO, has led to a reduced formation energy of PTO/Nb:STO interface by 0.32 eV relative to that of PTO/STO interface, in which the charge density is insufficient for a complete polarization screening (a [STO]_5.5_/[PTO]_5_ slab with an in-plane c($\sqrt{2}\times \sqrt{2}$) unit cell was used for the geometric structure of interface). According to Eq. 1, such lowered interface formation energy would make the wetting condition of PTO on Nb:STO accessible and would be beneficial for layer-by-layer epitaxial growth^16^. Meanwhile, the negative bound charges on the PTO surface could be compensated by adsorbing charged molecular species from the solution^20^. As a result, a low-temperature solution epitaxy of ferroelectric oxide films may be feasible by engineering the electronic polarization screening at the interface using the substrates with desired *n*-type doping concentration (Fig. 1a).

## Results and discussion

### Solution epitaxial growth of ferroelectric oxide films driven by polarization screening

To verify this experimentally (see Methods for more details), single-crystal (100)-oriented Nb:STO substrates (1 cm × 1 cm) with various Nb doping concentrations (0, 0.05, 0.5, 0.7, and 1 wt%) were selected to examine the growth of PTO films by a low-temperature hydrothermal method (100–200 ^o^C, below the *T*_c_ of PTO), where the ferroelectric polarization of PTO remains available. With *n*-type Nb doping, electrons become the majority carrier in Nb:STO; the conductivity and electron concentration are significantly enhanced by several orders of magnitude (Supplementary Table 1). In principle, the electron concentration of ≈10^20^ cm^−3^ is necessary for completely screening the bound charges at the positive polar surface of PTO^29^. According to Supplementary Table 1, such electron concentration can be provided by 0.7 wt% Nb:STO. As expected, a continuous and smooth PTO film epitaxially grows on 0.7 and 1 wt% Nb:STO substrates, whereas the films with pores and rough morphology can be observed on STO substrates with an Nb doping concentration below 0.7 wt% (Supplementary Fig. 2).

**Fig. 2:**
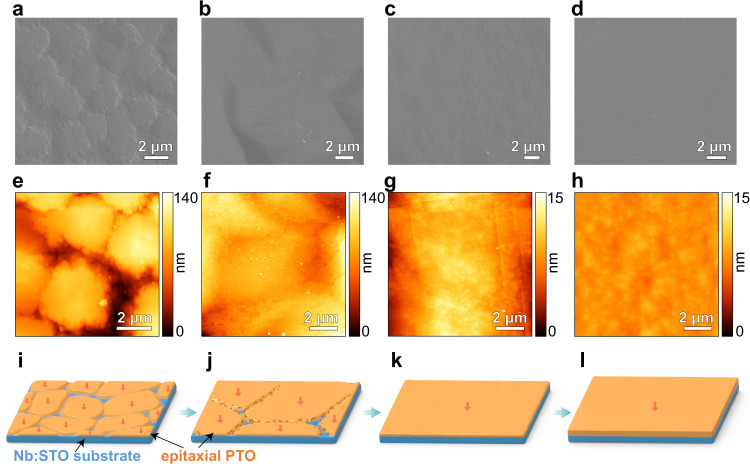
A two-dimensional layer-by-layer-like growth of PTO film on Nb:STO substrate. **a**–**h** SEM images (**a**–**d**) and AFM topography images (**e**–**h**) of PTO films obtained by hydrothermal synthesis for 2.5 h (**a**, **e**), 3 h (**b**, **f**), 4 h (**c**, **g**), and 12 h (**d**, **h**), respectively. **i**–**l** Schematic illustrations of the two-dimensional layer-by-layer-like growth of PTO film on Nb:STO substrate, arrows in each PTO crystal denote their uniform downward polarization direction towards the substrate.

The PTO film (epitaxially grown on 0.7 wt% Nb:STO substrate) was characterized to be a single-crystal tetragonal structure by synchrotron radiation X-ray Diffraction (Supplementary Fig. 3a, b), where the unit cell parameters *a* and *c* are 3.902 and 4.152 Å, respectively, close to those of perovskite tetragonal PTO (JCPDS #70-0746). In particular, the presence of four peaks at an interval of exactly 90° in the *ϕ* scan (Fig. 1b) further supports the single-crystal character of PTO film. A cross-sectional scanning transmission electron microscopy (STEM) image shows a film thickness of ≈530 nm and a coherent and sharp interface with the Nb:STO substrate (Fig. 1c). In addition, piezoelectric force microscopy (PFM) results demonstrate that the PTO film adopts a single-domain structure (Fig. 1d) and exhibits typical ferroelectric and piezoelectric characteristics (Supplementary Fig. 3c, d). Furthermore, a time-dependent morphology evolution of PTO film on Nb:STO has been systematically shown in Fig. 2. Figure 2a shows an SEM image of PTO hydrothermally synthesized for 2.5 h, where two-dimensional crystals homogeneously grow on the substrate with a saturated thickness of ≈80 nm and a lateral size of ≈3–4 μm, determined by atomic force microscopy (AFM, Fig. 2e). As the reaction time prolonged, these two-dimensional PTO crystals coarsen to a continuous film that subsequently undergoes a layer-by-layer-like growth (Fig. 2b–d). Correspondingly, the film thickness increases linearly (Supplementary Fig. 4) and the surface morphology becomes smoother in a range of 3–12 h, where the root mean squared roughness of the PTO surface reduces from 17.56 to 0.55 nm (Fig. 2f–h). Based on the above experimental results, we propose a growth process model of the film by a schematic in Fig. 2i–l. Owing to the electronic polarization screening at the interface, a reduced interface energy can drive two-dimensional nucleation and growth of PTO crystals in a local and homogeneous manner (Fig. 2i). Every crystal has a large lateral-thickness ratio of ≈50 with a downward polarization pointing to the substrate. Subsequently, guided by the wetting condition and the continuous raw materials supply from the solution, the crystals further grow in-plane by a ripening process until a continuous single-crystal film is formed, adopting a single-domain structure (Fig. 2j–l). We argue that the uniform polarization direction in every two-dimensional crystal would be beneficial for the growth of the single-crystal and single-domain film.

**Fig. 3:**
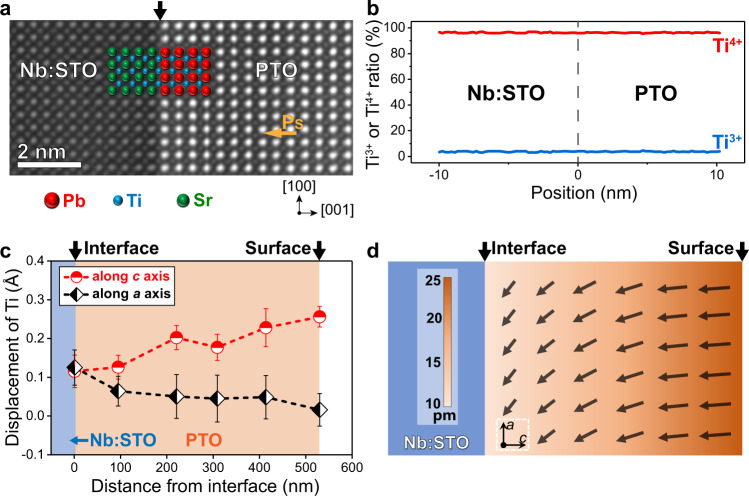
Characterization of PTO/Nb:STO interfacial structure and the polarization gradient throughout PTO film. **a** Atomic-level cross-sectional HAADF-STEM image of PTO/Nb:STO. **P**_**S**_ denotes the spontaneous polarization of PTO. **b** According to a series of Ti-*L*_2,3_ spectra across the PTO/Nb:STO interface in Supplementary Fig. 5b, Ti^3+^ and Ti^4+^ content are plotted as a function of the position away from the interface, where position zero represents the interface. **c** Experimentally extracted off-center displacements of the Ti ions as a function of the distance from the interface using cross-sectional HAADF-STEM images according to Supplementary Fig. 8. Error bars are indicative of the standard deviations (s.d.) of the displacements. **d** Schematic illustration of the polarization gradient throughout PTO film. The displacement magnitude of the Ti ions along the *c-*axis is depicted as shown in the color bar.

A high-angle annular dark-field STEM (HAADF-STEM) image reveals a well-defined and atomically sharp interface without detectable elemental diffusion (Fig. 3a). In tetragonal perovskite PTO, the spontaneous polarization can be determined by the off-center displacements of the Ti ions^9^. In Fig. 3a, all Ti ions in PTO shift upward relative to the center of the four nearest Pb ion columns, confirming a downward polarization direction. To explore the electronic states near the interface, core loss spectra from electron energy loss spectroscopy (EELS) were acquired across the interface (Supplementary Fig. 5). In PTO/Nb:STO heterostructure, the Ti^3+^ content (the detectable limit of this method ~4%) across the interface has no obvious change except for small fluctuations within a reasonable error range (Fig. 3b). Owing to the sufficient electrons in Nb:STO substrate, the polarization screening of PTO film can be accomplished, leading to a single-domain structure. By contrast, there is a significantly increased amount of Ti^3+^ near the PTO/STO interface accompanied by the existence of 180° domains (Supplementary Fig. 5g, h), implying relatively high interface energy (Supplementary Fig. 1b). The presence of 180° domain structure in ferroelectrics is a typical mechanism for polarization screening^20^.

Experimentally, either atomic reconstruction or elemental diffusion is not detectable on both sides of the interface in Fig. 3a, where the displacement of Ti ions in Nb:STO side was not observed. Hence, the electron concentration in the substrate is proposed to play a major role in altering the growth mode of PTO from an island to a layer-by-layer-like growth at low temperatures (Supplementary Fig. 6). This understanding may explain the observed island growth of PTO on undoped STO substrates despite the very small lattice mismatch between them^4,19^. More generally, this electronic polarization screening strategy can also be applied to drive a low-temperature solution epitaxy of other perovskite ferroelectric oxide films, such as bismuth ferrite (BiFeO_3_, BFO), potassium niobate (KNbO_3_, KNO) and lead zirconate titanate Pb(Zr_x_,Ti_1-x_)O_3_ (x = 0.05–0.20) (Supplementary Fig. 7). Moreover, it has been illustrated that the compensation of the polarization charges from the finite electronic screening length of metallic electrodes may be imperfect^30^. Therefore, other screening mechanisms, including interfacial octahedral linking or ionic screening^26,31^ could also make a minor contribution to our PTO/Nb:STO system.

### Investigation on polarization gradient of PTO film

Surprisingly, the films exhibit a fascinating polarization gradient from the interface to the surface (Fig. 3d), determined by the off-center displacements of the Ti ions^32^, both out-of-plane (*c*-axis) and in-plane (*ab-*plane) (Fig. 3c). The displacements were derived at different distances from the interface by using atomic-level HAADF-STEM images (Supplementary Fig. 8). From Fig. 3c, the out-of-plane displacements of Ti ions are suppressed near the interface and gradually increase when moving away from the interface throughout the whole film thickness; the displacements of surface Ti ions (≈0.25 Å) remain lower than those of bulk PTO (>0.3 Å)^26,27^. In addition, in-plane displacements of Ti ions near the interface have been observed, revealing the existence of a monoclinic phase^31^ of PTO film near the interface. It has been theoretically predicted that a polarization gradient ${{{{{\boldsymbol{\nabla }}}}}}{{{{{\bf{P}}}}}}$ could be generated in ferroelectrics by a coupling of composition gradient ${{{{{\boldsymbol{\nabla }}}}}}{{{{{\bf{c}}}}}}$ or strain gradient ${{{{{\boldsymbol{\nabla }}}}}}{{{{{\boldsymbol{\delta }}}}}}$ or temperature gradient$\,{{{{{\boldsymbol{\nabla }}}}}}{{{{{\bf{T}}}}}}$ with the polarization vector during the growth process: ${{{{{\boldsymbol{\nabla }}}}}}{{{{{\bf{P}}}}}}{{{{{\boldsymbol{\propto }}}}}}{{{{{\boldsymbol{\nabla }}}}}}{{{{{\bf{c}}}}}}{{{{{\boldsymbol{,}}}}}}\,{{{{{\boldsymbol{\nabla }}}}}}{{{{{\boldsymbol{\delta }}}}}}{{{{{\boldsymbol{,}}}}}}{{{{{\boldsymbol{\nabla }}}}}}{{{{{\bf{T}}}}}}$^33^. The strain and temperature gradients, however, are negligible in our system because of a very low lattice mismatch and solution environment. Consequently, we argue that a composition gradient is possibly responsible for the polarization gradient of these PTO films. Experimentally, the composition gradient is determined by time-of-flight secondary-ion mass spectra (TOF-SIMS), where the Pb/Ti ratio (calculated by dividing the Pb and Ti ion signals) is reduced by ~3.5% at the interface relative to the surface of PTO film (Supplementary Fig. 9). This composition gradient is expected to account for the formation of the structure gradient characterized by nanobeam electron diffraction analysis^34^ (Supplementary Fig. 10) and the polarization gradient, where lattice parameter *c* gradually increases from 4.111 ± 0.021 Å (near the interface) to 4.152 ± 0.008 Å (near the surface), as the lattice parameter *a* decreases from 3.924 ± 0.016 Å to 3.905 ± 0.010 Å. Recently, an inhomogeneous composition distribution, such as Zr distribution in Ti-rich Pb(Zr,Ti)O_3_ single crystals, has been found to generate Néel-like domain walls, where a monoclinic phase was identified to be critical for the polarization rotation^35^. Such achievement encourages us to further explore possible origin of the composition gradient and the monoclinic phase of PTO films.

In order to figure out the origin of the composition gradient, we performed a solution growth of PTO film on Nb:STO substrate for only 1 h (Supplementary Fig. 11). The products have been characterized to be composed of crystalline PbTi_0.8_O_2.6_ (JCPDS #49-0863) and PbTiO_3_ (JCPDS #70-0746) nanoparticles as well as a bare Nb:STO substrate (Supplementary Fig. 11a–c). It is reasonable to understand that the homogeneous nucleation of these nanoparticles could largely occur at the initial stage, where the concentration of dissolved raw materials in a closed hydrothermal system at 200 ^o^C was high to easily reach supersaturation. This would lead to a quick decrease in the concentration of Pb in solution at the initial stage (Supplementary Fig. 11d). Correspondingly, the heteroepitaxy of PTO on the substrate was suppressed kinetically at this stage (Supplementary Fig. 11a, c). As the reaction proceeds, the metastable PbTi_0.8_O_2.6_ nanoparticles gradually dissolved, leading to a slow increase of the concentration of Pb in solution (Supplementary Fig. 11d). The heteroepitaxy of PTO film on the substrate thus occurred when the condition was close to equilibrium. Pb vacancies have been predicted to be spontaneously formed during the crystal growth of PTO^36^. Hence, Pb vacancies were more easily generated at the beginning of the heteroepitaxial growth of the PTO film in our system. As the heteroepitaxy continues, the formation of Pb vacancies gradually becomes more difficult due to an increased concentration of Pb in the solution. In the end, the concentration of Pb would saturate to a certain value because of the dissolution balance among the PTO nanoparticles, films, and the solution.

It has been discovered that a monoclinic phase exists in the PZT system with a poor Zr concentration (10%), which is probably originated from inhomogeneous composition distribution^31^. Inspired by this work, we argue that a monoclinic phase of PTO film near the interface could arise from the existence of Pb vacancies. In addition, the partial breaking of Pb-O covalent bonds may not be beneficial to the stability of the tetragonal phase^37^, favoring the formation of the monoclinic phase. Subsequently, the deceased Pb vacancy from the interface to the surface generated during the epitaxial growth would give rise to a transition from monoclinic to tetragonal phase and, thus, a polarization gradient.

### Photovoltaic properties and mechanism

**Fig. 4:**
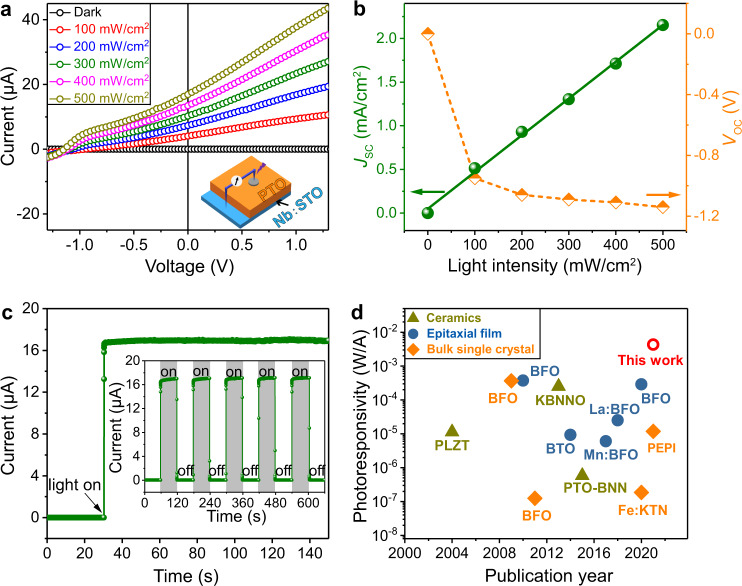
Photovoltaic properties of PTO film with the polarization gradient. **a** *I-V* characteristics of PTO film under the illumination of different *I*_light_ from a 375 nm ultraviolet laser as well as dark current. Inset shows a schematic of the setup for photovoltaic measurements. **b** *J*_SC_ and *V*_OC_ as a function of *I*_light_. The green solid line is fitted to the *J*_SC_ data. **c** Steady-state short-circuit current as a function of time at zero bias when *I*_light_ of 500 mW/cm^2^ is turned on. Inset shows the short-circuit current response to the switching of a light on and off at zero bias. **d** Photoresponsivity in the bulk phase of PTO film (red circle, this work), compared with previously reported ferroelectrics (Listed in Supplementary Table 2 in the order of publication year).

One should note here that the polarization direction of PTO film is difficult to be switched by PFM (Supplementary Fig. 3f), which could be associated with the built-in electric field generated by the polarization gradient^6,32,33^. Such a built-in electric field can separate and transport the photogenerated carriers, similar to that in the depletion region of the *p-n* junction (Supplementary Fig. 12). Accordingly, the photovoltaic properties of PTO film have been investigated (inset of Fig. 4a shows the device structure). Since the bandgap of PTO has been determined to be ≈3.0 eV^38^, a linearly polarized laser with a wavelength of 375 nm and power intensity from 0–500 mW/cm^2^ was employed. Ag film (area: ~0.00785 cm^2^, thickness: ~40 nm) was deposited on the PTO surface as a top electrode, and the Nb:STO substrate acted as a bottom electrode.

Figure 4a displays the current-voltage (*I-V*) characteristics of the grown PTO film under the illumination of different light intensities (*I*_light_). The *I-V* curves obtained in a voltage range from −1.3 to 1.3 V manifested a near-linear behavior. Moreover, the short-circuit current density (*J*_SC_) is linearly proportional to *I*_light_, implying that the number of photogenerated carriers is determined by *I*_light_. As a comparison, PTO film under dark conditions shows negligible response (Fig. 4a, black line). Meanwhile, the open-circuit voltage (*V*_OC_) first increases with *I*_light_ and then reaches a saturation value of ≈1.15 V (Fig. 4b). When *I*_light_ of 500 mW/cm^2^ illuminated PTO surface under zero-bias condition, a steady-state short-circuit current of ≈16.9 μA (*J*_SC_ ≈ 2.153 mA/cm^2^) has been obtained (Fig. 4c), which can be well controlled by turning the light on and off (inset of Fig. 4c). Furthermore, the short-circuit current and *V*_OC_ of the device can be reversed by a switched polarization (Supplementary Fig. 13).

Figure 4d shows the reported photoresponsivity in the bulk phase of ferroelectrics over the past two decades (Supplementary Table 2). In this work, the obtained *J*_SC_ that arises from the photovoltaic effect of PTO film has been determined to be 2.153 mA/cm^2^, higher than those of ferroelectrics by two orders of magnitude. Considering the different *I*_light_, the photoresponsivity (*J*_SC_*/I*_light_) of PTO has been determined to be 4.306 × 10^−3^ A/W. This value is still one order of magnitude higher than the studied ferroelectrics and comparable with the calculated maximum shift current in other polar compounds, such as LiAsS_2_, LiAsSe_2_, and NaAsSe_2_^39^. One may argue that Schottky barriers on either side of PTO film can also contribute to the photovoltaic current. To address this issue, Au, Ag, and Pt top electrodes with different work functions *W*_F_, have been deposited on PTO film for photovoltaic measurements, and the results certainly exclude the major contribution of the Schottky barrier (Supplementary Fig. 14a, b). In addition, the effective distance of the barrier at PTO/Nb:STO interface has been determined to be ≲50 nm for generating the photovoltaic current^40^. In contrast, a large photovoltaic current remains available even when the thickness of PTO films is up to 1 μm (Supplementary Fig. 14c, d). Furthermore, considering the single-domain structure of PTO film, the contribution of the domain wall to the photovoltaic effect^41^ could be excluded.

The shift current theory has been widely studied and it is an important part of the bulk photovoltaic effect in tetragonal ferroelectric BaTiO_3_ and PTO^42^, and recent work showed that the ballistic current mechanism could give a similar magnitude as shift current^43^. To estimate their contributions, the shift current density was calculated for various displacements of Ti ions under a wide range of wavelengths of incident light (310–496 nm, 100 mW/cm^2^) (Supplementary Fig. 15). However, the largest shift current density has been determined to be 0.018 mA/cm^2^, much lower than the experimental *J*_SC_ value of 0.513 mA/cm^2^ measured under *I*_light_ of 100 mW/cm^2^ in Fig. 4b. This result implies that a shift current or ballistic current cannot make the dominant contribution to the photovoltaic current in Fig. 4. At this stage, we argue that such colossal photovoltaic current probably arises from the polarization gradient and thus built-in electric field within PTO film.

As a fundamental issue, the polarization screening between ferroelectric films and substrates is generally discussed to be crucial for the polarization stability, domain structure, and switching dynamics as well as device performance^2,20,25^. In this work, we propose that an electronic polarization screening is able to tune the interface energy and thus enable a solution route to high-quality epitaxial growth of single-crystal and single-domain ferroelectric oxide films at low temperatures. Moreover, this solution epitaxy allows to obtain a polarization graded film with the remarkable photovoltaic current. We anticipate that our work will motivate a search for the photo-induced effects in gradient ferroelectric films, such as photovoltaic and photostrictive effects^44,45^, prior to novel sensor and detector applications.

## Methods

### Materials

Lead nitrate (Pb(NO_3_)_2_, ≥99.0%), tetrabutyl titanate (TBOT, ≥98.0%), potassium hydroxide (KOH, ≥85.0%), acetone (≥99.5%), and ethanol (≥99.7%) were purchased from Sinopharm Chemical Reagent Co. Ltd. and used without further purification. All cubic STO or Nb:STO single-crystal substrates with dimensions 10 × 10 × 0.5 mm^3^ and (100) orientation were obtained from Shenyang Baijujie Corporation, and they were two-sided polished.

### Film preparation

PTO films were synthesized by a hydrothermal method as follows: Pb(NO_3_)_2_ and TBOT were used as starting materials. KOH was used as a mineralizer. STO or Nb:STO substrate was cleaned ultrasonically with acetone, ethanol and deionized water before using and then placed in a Teflon holder for drying. The Pb/Ti molar ratio was set at 1.25:1 and the concentration of KOH was 6 M. KOH was first dissolved in deionized water, 1.37–1.71 g of TBOT was then added in the aqueous solution, Pb(NO_3_)_2_ solution was finally dropwise added under vigorous stirring. The volume of the solution reaches 80% of the autoclave (50 ml). After 2 h of continuous stirring at room temperature, STO or Nb:STO substrate was placed in the solution of the autoclave before hydrothermal treatment under 200 ^o^C for 3–12 h. STO or Nb:STO substrate was maintained horizontally 15 mm above the bottom of the autoclave. The resultant products were washed with water and ethanol several times and subsequently dried at 60 ^o^C in the air for further characterization.

### Characterization

The structures of the sample were characterized by X-ray diffraction (XRD, Rigaku D/max-RA, Cu *Kα* radiation, *λ* = 1.5406 Å), scanning electron microscopy (SEM, Hitachi SU-70), and scanning probe microscopy (SPM, Asylum Research, Cypher S and MFP-3D). The synchrotron radiation XRD was carried out at the beamline 15U1, Shanghai synchrotron radiation facility (SSRF), China. The X-ray wavelength was 0.6199 Å. The X-ray beam was focused down to 3 × 3 μm^2^ by a Kirkpatrick–Baez mirror. The detector was a Mar165 charge-coupled device (CCD). X-ray reciprocal-space mapping (RSM) was performed on Bruker D8 Discover Diffractometer, equipped with a High-brilliance rotational Cu X-ray source, DUO detectors of Scintillation counter, and LynxEye detector. The thin-film samples were measured in a grazing-incidence mode with an exposure time of 5 s for each pattern. An integrated focused ion beam (FIB, Helios nanolab 600) and SEM (Hitachi S4700) was used to extract thin-film lamellae from the bulk PTO/Nb:STO sample, Pt and Au layers were deposited on the film surface to protect from damage and increase the conductivity, respectively. Subsequent cross-sectional observations of the film were carried out using transmission electron microscopy (TEM, FEI Tecnai G2 F20 S-TWIN) and aberration-corrected transmission electron microscopy (FEI Titan G2 80-200 Chemi STEM). Nanobeam electron diffraction was carried out using aberration-corrected transmission electron microscopy (FEI Titan G2 60-300). The depth profiling of the ion signals throughout the films were characterized by time-of-flight secondary-ion mass spectrometry (TOF-SIMS, Iontof TOF.SIMS 5). The depth profiling by TOF-SIMS is in positive polarity, where a 30 keV primary-ion beam of Bi_1_^+^ first impacted the film surface, followed by a 2 keV sputtering beam of O_2_. The area of depth profiling is 200 μm × 200 μm.

### Interfacial electronic structure calculations

The first-principle calculations for PTO/STO (Nb:STO) interface were performed based on the density-functional theory (DFT) as implemented in the Vienna ab initio package (VASP)^46^. The projector augmented wave basis^47^ sets and the generalized gradient approximation of Perdew, Burke, and Ernzerhof (PBEsol)^48,49^ to the exchange−correlation functional were chosen. Due to the non-negligible electron correlation, the on-site Hubbard *U* was employed to correct the localization of the *d*-electrons within DFT + *U* method^50^: *U* − *J* = 3 eV/2 eV for 3*d* electrons of Ti/Nb respectively. To capture the characteristics of the interface, we adopted the inplane $\sqrt{2}\times \sqrt{2}$ [SrTiO_3_]_5.5_/[PbTiO_3_]_5_ slab sandwiched by 25 Å vacuum. The atomic positions of PTO were fixed to keep the physical polarization except for the interfacial area. The force criteria were set to be 0.02 eV/ Å and energy difference was converged to 10^−4^ eV. The energy cutoff was set as 400 eV for all calculations and 10 × 10 × 1 *k*-points were utilized to sample the Brillouin zone. Differential charge density was defined as follows:

$$\triangle \rho={\rho }_{{{{{{\rm{total}}}}}}}-{\rho }_{{{{{{\rm{PTO}}}}}}}-{\rho }_{{{{{{\rm{STO}}}}}}}$$

in which, ${\rho }_{{{{{{\rm{PTO}}}}}}}$ or ${\rho }_{{{{{{\rm{STO}}}}}}}$ are the charge density of PTO or STO, respectively, and ${\rho }_{{{{{{\rm{total}}}}}}}$ corresponds to the total charge density of the slab.

The 10% Nb doping was applied in this model due to computational complexity caused by a huge amount of unit cells if 0.7 wt% Nb doping was calculated.

### Polarization measurement

In this work, PTO has a tetragonal structure (Supplementary Fig. 3). Both the oxygen octahedra and the Ti ions have displacements from the center of the Pb ions tetragonal cell that gives rise to the spontaneous polarization. The displacements of Ti ions can be used to determine the polarizations of PTO unit cells^9^. Hence, the Ti ions shift relative to the center of the four nearest Pb ion columns were measured in cross-sectional HAADF-STEM-based images of extracted thin-film lamellae by Digital Micrograph software.

### Photovoltaic measurement

Photovoltaic properties of PTO films were collected using optical units from Thorlabs and source measure units (Keithley 6450). Ag electrode (area: ~0.00785 cm^2^, thickness: ~40 nm) was deposited on PTO surface by direct current (dc) sputtering (SD-3000). The samples were illuminated with light from 375-nm laser (MDL-III-375). The polarization switching is realized by applying 30 V reverse dc bias on PTO film.

### Shift current simulations

We calculated the shift current responding to a 100 mW/cm^2^ wide-range wavelengths (310–496 nm) of incident light from first-principles simulations as developed in ref. ^42^ for PTO with experimental parameters. In particular, instead of using the structure for normal tetragonal PTO where the displacements of Ti ions are along *c*-axis only with a fixed magnitude, here we allow the displacements of Ti ions within the *ab*-plane with various magnitude. We performed two types of calculations, one in which Ti can move along *a*- and *c*-axis, whereas in the other calculation, Ti can move along *a*-, *b*-, and *c*-axis. In each type of calculation, the lattice parameters are obtained from ref. ^51^, and the oxygens are fixed in their relaxed positions. Four different sets of displacement magnitude for Ti ions were chosen as indicated in Supplementary Fig. 15a, and Pb will also move along *c*-axis accordingly so that Ti and Pb will achieve the high-symmetry positions at the same time. DFT was performed with norm-conserving pseudopotentials^52^ on an 8 × 8 × 8 k-grid to obtain the charge density, and denser *k*-grids are used to obtain the required ingredients such as momentum matrix and Berry connections^42^.

### Supplementary information

Editorial Assessment Report

Supplementary Information

## Data Availability

The data that support the findings of this study are available from the corresponding author upon reasonable request.

## Code availability

The codes that support the findings of this study are available from the corresponding author upon reasonable request.
