# Addendum: Solution epitaxy of polarization-gradient ferroelectric oxide films with colossal photovoltaic current

**DOI:** 10.1038/s41467-024-47238-z

**Published:** 2024-05-08

**Authors:** Chen Lin, Zijun Zhang, Zhenbang Dai, Mengjiao Wu, Shi Liu, Jialu Chen, Chenqiang Hua, Yunhao Lu, Fei Zhang, Hongbo Lou, Hongliang Dong, Qiaoshi Zeng, Jing Ma, Xiaodong Pi, Dikui Zhou, Yongjun Wu, He Tian, Andrew M. Rappe, Zhaohui Ren, Gaorong Han

**Affiliations:** 1 State Key Laboratory of Silicon and Advanced Semiconductor Materials, School of Materials Science and Engineering, Zhejiang University, Hangzhou, 310027 China; 2 Center of Electron Microscope, School of Materials Science and Engineering, Zhejiang University, Hangzhou, 310027 China; 3 Department of Chemistry, University of Pennsylvania, Philadelphia, PA 19104-6323 USA; 4 Oden Institute for Computational Engineering and Sciences, The University of Texas at Austin, Austin, Texas 78712 USA; 5 Key Laboratory for Quantum Materials of Zhejiang Province, Department of Physics, School of Science, Westlake University, Hangzhou, 310024 China; 6 Zhejiang Province Key Laboratory of Quantum Technology and Device, Department of physics, Zhejiang University, Hangzhou, 310027 China; 7 Center for High Pressure Science and Technology Advanced Research, Shanghai, 201203 China; 8 State Key Lab of New Ceramics and Fine Processing, School of Materials Science and Engineering, Tsinghua University, Beijing, 100091 China; 9 Institute of Advanced Semiconductors & Zhejiang Provincial Key Laboratory of Power Semiconductor Materials and Devices, ZJU-Hangzhou Global Scientific and Technological Innovation Center, Zhejiang University, Hangzhou, 311215 China; 10 Research Center for Intelligent Sensing, Zhejiang Lab, Hangzhou, 311100 China

**Keywords:** Ferroelectrics and multiferroics, Synthesis and processing

Addendum to: *Nature Communications* 10.1038/s41467-023-37823-z, published online 24 April 2023

In this article, we reported a solution epitaxy of ferroelectric oxide films, focusing on single-crystal film of PbTiO_3_ on single-crystal Nb:SrTiO_3_ substrate. We would like to provide additional information to further support its epitaxy and single-crystal character by electron back-scattered diffraction (EBSD) and rocking curve analysis. Figure 1a presents a typical EBSD band contrast map of PbTiO_3_ film in a relatively large area, and the uniform color of the inverse pole figure (IPF) map in the Z direction (IPF Z in Fig. 1b) demonstrates that PbTiO_3_ film is a single crystal. Furthermore, the fullwidth at half maxima (FWHM) of the rocking curve (Fig. 1c) scanned around the (001) diffraction peak is only 0.028°, indicating a high-quality epitaxy of PbTiO_3_ film on the substrates.
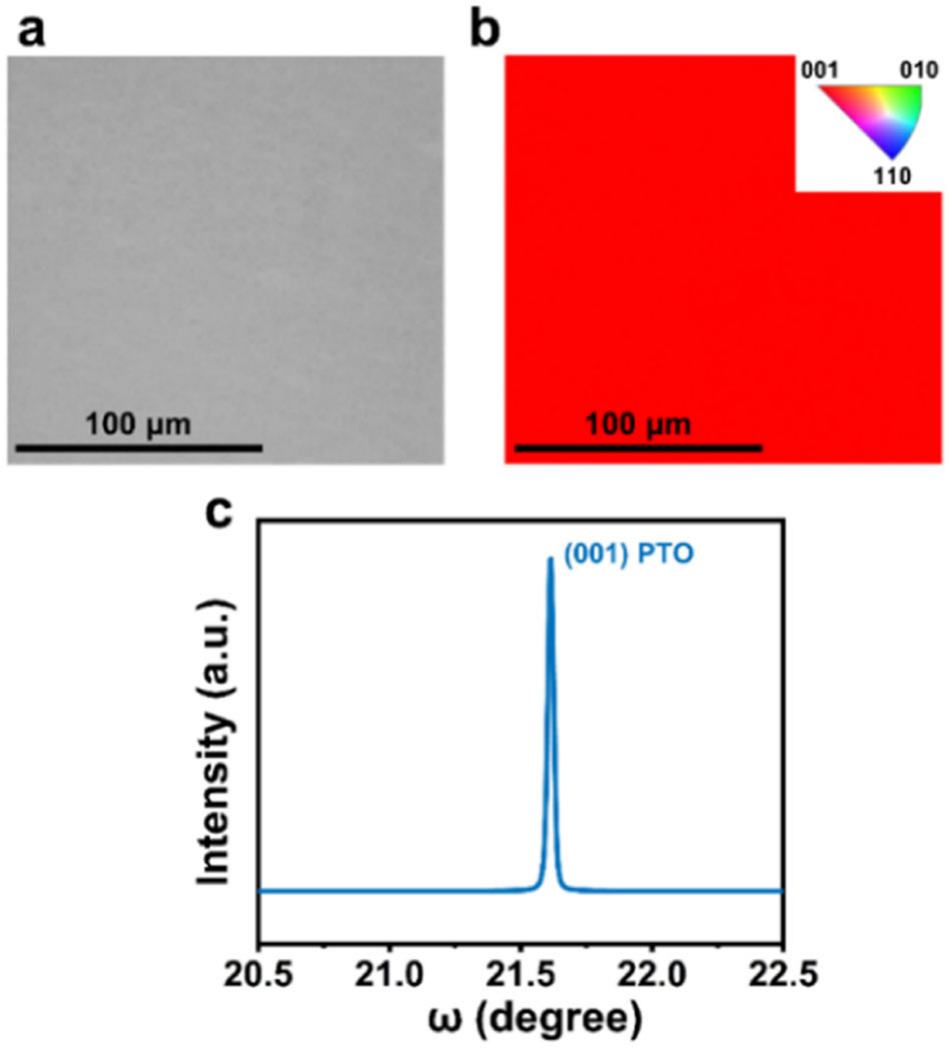


**Fig. 1 EBSD and rocking curve analysis of PbTiO**_**3**_ **film on (100) Nb(0.7wt%): SrTiO**_**3**_ **substrate. a** EBSD band contrast image. **b** corresponding EBSD IPF Z direction color map. **c** rocking curve of PTO film (001) peak.

