# Supplementary material for: Solution epitaxy of polarization-gradient ferroelectric oxide films with colossal photovoltaic current
Source: Nat Commun. 2023 Apr 24;14:2341. doi: 10.1038/s41467-023-37823-z (PMC10126087; doi:10.1038/s41467-023-37823-z)
Supplement: Supplementary file 1 — Editorial Assessment Report [file 41467_2023_37823_MOESM1_ESM.pdf]

nature portfolio

GUIDED OPEN ACCESS

nature physics | nature communications | communications physics

Contents of this report

1. **Manuscript details**: overview of your manuscript and the editorial team.
2. **Review synthesis**: summary of the reviewer reports provided by the editors.
3. **Editorial evaluations**: personalized evaluation and recommendation from all 3 journals.
4. **Annotated reviewer comments**: the referee reports with comments from the editors.
5. **Open research evaluation**: advice for adhering to best reproducibility practices.

About the editorial process

Because you selected the **Nature Portfolio Guided Open Access** option, your manuscript was assessed for suitability in three of our titles publishing high-quality work across the spectrum of physics research: ***Nature Physics***, ***Nature Communications***, **and** ***Communications Physics***. More information about Guided Open Access can be found here.

Collaborative editorial assessment

Your editorial team discussed the manuscript to determine its suitability for the Nature Portfolio Guided OA pilot. Our assessment of your manuscript takes into account several factors, including whether the work meets the **technical standard** of the Nature Portfolio and whether the findings are of **immediate significance** to the readership of at least one of the participating journals in the Nature Portfolio Guided Open Access physics cluster.

Peer review

Experts were asked to evaluate the following aspects of your manuscript:

- **Novelty** in comparison to prior publications;
- **Likely audience** of researchers in terms of broad fields of study and size;
- **Potential impact** of the study on the immediate or wider research field;
- **Evidence** for the claims and whether additional experiments or analyses could feasibly strengthen the evidence;
- **Methodological detail** and whether the manuscript is reproducible as written;
- Appropriateness of the **literature review**.

Editorial evaluation of reviews

Your editorial team discussed the potential suitability of your manuscript for each of the participating journals. They then discussed the revisions necessary in order for the work to be published, keeping each journal's specific editorial criteria in mind.

Journals in the Nature portfolio will support authors wishing to transfer their reviews and (where reviewers agree) the reviewers' identities to journals outside of Springer Nature.
If you have any questions about review portability, please contact our editorial office at guidedoa@nature.com.

Manuscript details

Tracking number	Submission date	Decision date	Peer review type
GUIDEDOA-21-00328	Nov 17, 2021	Jan 10, 2022	Single-blind

Manuscript title

Solution epitaxy of polarization-gradient ferroelectric oxide films with colossal photovoltaic current

Preprint: N/A

Author details

Gaorong Han

Affiliation: Zhejiang University

Editorial assessment team

**Primary editor**	**Andrea Taroni**<br>Home journal: ***Nature Physics***<br>ORCID: 0000-0001-9550-5754<br>Email: **a.taroni@nature.com**
**Other editors consulted**	**Jiajun Zhu**<br>Home journal: ***Nature Communications***<br>ORCID:<br><br>**Saleem Denholme**<br>Home journal: ***Communications Physics***<br>ORCID: 0000-0001-6017-0819
**About your primary editor**	Andrea joined Nature Physics in 2014, having previously worked for Nature Communications and Nature Materials. A graduate of University College London, he completed his PhD in statistical physics. Following a short stint at the École Normale Supérieure de Lyon in France, he undertook postdoctoral work at Uppsala University in Sweden, investigating the dynamics of low-dimensional magnetic systems. Andrea is based in our London office.

Editorial assessment and review synthesis

**Editor's summary and assessment**	The authors report the fabrication of single-domain ferroelectric oxide films on Nb-doped SrTiO3 single-crystal substrates by solution reaction under a low temperature of ~200℃. They perform a comprehensive set of characterisation experiments, and report a range of appealing qualities, most notably a large photovoltaic response.
**Editorial synthesis of reviewer reports**	The reviewers confirm our initial editorial assessment in identifying the potential of this study, and recognise these results seem likely to stimulate the attention of other specialists interested in the fabrication of high-quality ferroelectric films.<br><br>However, the reviewers also raise a number of concerns that would need to be convincingly addressed in order for the manuscript to be given consideration at either *Nature Communications* or *Communications Physics*.<br><br>In particular, for further consideration at *Nature Communications* the authors will need to extend their discussion on the parameters and protocols used for the formation of the films, in order to make the results more reproducible. Moreover, they will also need to provide more convincing arguments to demonstrate that the effect they observe is indeed due to electron screening, rather than a change in lattice parameter. Finally, they will also need to show better support for the phase structure of PTO, as well as on the nature of the domains and domain wall structures, and provide additional experimental evidence to support the observation of the claimed polarization gradient.<br><br>For further consideration at *Communications Physics*, the authors would not necessarily be expected to perform the additional experiments to confirm the observation of the polarization gradient, but all efforts to confirm the reproducibility of these observations should be done.

Editorial evaluations

Nature Physics<br><br>Revision not invited	This study may well attract interest within the specialist community working on the fabrication and characterization of ferroelectric films, but as the appeal beyond that community appears to be limited, we do not feel the study matches the criteria for further consideration at *Nature Physics*.
Nature Communications<br><br>Major revisions	While we acknowledge the influence this study will likely have in the area of ferroelectrics, we require additional data and analysis in order to consider this manuscript further at *Nature Communications*.
Communications Physics<br><br>Minor revisions	We also appreciate the likely influence of this study among fellow specialists, and are happy to leave a more detailed investigation on the mechanism of crystal growth to a subsequent study. However, for further consideration at *Communication Physics*, we do require the referees' concerns over reproducibility to be convincingly addressed.

nature portfolio

GUIDED OPEN ACCESS

nature physics | nature communications | communications physics

Next steps

**Editorial recommendation 1:**	Our top recommendation is to revise and resubmit your manuscript to *Nature Communications.* We feel the additional experiments required to achieve this would result in a compelling study.
**Editorial recommendation 2:**	You may also choose to revise and resubmit your manuscript to *Communications Physics.* This option might be best if the requested experimental revisions are not feasible at this time.
**Note**	As stated on the previous page *Nature Physics* is not inviting a revision. Please keep in mind that the journal will not be able to consider any appeals of their decision through Guided Open Access.

Revision

To follow our recommendation, please upload the revised manuscript files using **the link provided in the decision letter**. Should you need assistance with our manuscript tracking system, please contact Adam Lipkin, our Nature Portfolio Guided OA support specialist, at guidedOA@nature.com.

Revision checklist

- ☐ Cover letter, stating to which journal you are submitting
- ☐ Revised manuscript
- ☐ Point-by-point response to reviews
- ☐ Updated Reporting Summary and Editorial Policy Checklist
- ☐ Supplementary materials (if applicable)

Submission elsewhere

If you choose not to follow our recommendations, you can still take the reviewer reports with you.

Option 1: Transfer to another Nature Portfolio journal
Springer Nature provides authors with the ability to transfer a manuscript within the Nature Portfolio, without the author having to upload the manuscript data again. To use this service, **please follow the transfer link provided in the decision letter.** If no link was provided, please contact guidedOA@nature.com.

> *Note that any decision to opt in to In Review at the original journal is not sent to the receiving journal on transfer. You can opt in to In Review at receiving journals that support this service by choosing to modify your manuscript on transfer.*

Option 2: Portable Peer Review option for submission to a journal outside of Nature Portfolio
If you choose to submit your revised manuscript to a journal at another publisher, we can share the reviews with another journal outside of the Nature Portfolio if requested. You will need to request that the receiving journal office contacts us at guidedOA@nature.com. We have included editorial guidance below in the reviewer reports and open research evaluation to aid in revising the manuscript for publication elsewhere.

Annotated reviewer reports

The editors have included some additional comments on specific points raised by the reviewers below, to clarify requirements for publication in the recommended journal(s). However, please note that all points should be addressed in a revision, even if an editor has not specifically commented on them.

Reviewer #1 information	
**Expertise**	Inorganic materials chemistry, thin films, growth techniques
**Editor's comments**	Overall, this reviewer is positive. Nevertheless, they request a more detailed discussion regarding the influence of electron screening that you propose as an explanation for your observations.

Reviewer #1 comments	
**Section**	**Annotated Reviewer Comments**
**Remarks to the Author: Overall significance**	This is a very well written and thorough work on deposition of epitaxial complex oxides via the solution phase. Such an approach to obtain this type of quality is in itself rather spectacular and will undoubtedly reach attention. The fact that it is a technologically relevant material, makes it even better. The high quality is explained by shielding of surface charges. That is a plausible explanation. I am not fully convinced that it is proven here, but that is of minor importance in this setting. This paper will make a good contribution in this direction.
**Remarks to the Author: Impact**	I do believe this contribution will create debate and influence growth of complex oxides from solution phase. The magnitude of work and thorough analysis makes this paper worth reading and debating.
**Remarks to the Author: Strength of the claims**	The claims of high-quality work is highly convincing as supported by thorough structural characterization.<br><br>The reason for the high quality is not strictly evident. The authors have suggested the effect of free electrons that shield the polarization that otherwise would lead to domain structures. This is a plausible claim and one that I believe at this stage should be allowed to be as is. However, I would anticipate further investigations on attempts to perturb this by applying electrical fields or other types of substrates with free electrons but not necessarily that well matched in lattice. However, the magnitude of such work is large, and hence, something I recommend for someone to follow up on.

We ask the authors to take these criticisms on board, although we note that we do not expect them to perform additional experiments on different substrates in order to warrant further consideration at either *Nature Communications* or *Communications Physics.*

Specific minor comments:
Line 29: Suggest changing word from «complicate” to “different”. Can also use “complex”, however, they can also be complex in the gas-phase.

Line 88: Suggest to change “model” to “computational model”, since it was not clear that this paragraph was about computational approach until later into it.

Line 97: I cannot find equation 1

Lines 97, 99, 101: The words “would”, “could”, “is” must cohere. “is” can be replaced with “may be”.

Line 109: “<110> direction”: The orientation of the substrate must also be given for this experiment. I assume (100), but this is not given.

Line 110: What does figure c show? To me it is two grey slabs. I fail to see the meaning of this figure. I do acknowledge the text “coherent and sharp interface with the Nb:STO substrate (Fig. 1c).” but given that the substrate is flat to begin with, this does not surprise me. In other words, the image needs to be at a higher magnification to convince.

Line 115: “low-temperature hydrothermal” To me 100-200 C is not low temperature when it comes to hydrothermal. Suggest omitting “low-“

Line 157: When a film grows in an epitaxial manner as shown in Fig 2. I would expect anti-phase effects along the grain boundaries. Can the authors comment on this? Would such be an issue in this case?

Line 175: On one side Nb doping into STO provides free carriers, but it also provides a variation in unit cell lattice dimensions. How can the effects of these two be isolated for the effect of epitaxial growth seen here? In other words, I am impressed of the results, but not really convinced that it is due to electron screening rather than change in lattice parameter. I need some more arguments.

For further consideration at *Nature Communications*, the authors are encouraged to argue more convincingly (qualitatively and/or quantitatively) that the effect they observe is indeed due to electron screening, rather than a change in lattice parameter.

Line 195: Will Nb-doping have any effect on Ti3+ content?

	Line 218: I do not follow the logic: Theory predicts precipitation below 140 C, hence at 200 C it grows homogeneously, i.e. spontaneous nucleation.?? I do not follow.<br><br>Line 282: “even the” -> “even when the”<br><br>Line 408: Neither amounts or concentrations are given. Hence, this is not on a level that can be reproduced by others.<br><br>Line 694: How has the schematics of Pb concentration with time been obtained? What is the logic behind this evolution?
**Remarks to the Author: Reproducibility**	The procedures for formation of these films is way too crudely formulated to allow anyone to reproduce these. That part must be updated.<br><br>**The authors should extend their discussion on the parameters and protocols used for the formation of the films, in order to make the results more reproducible.**

Reviewer #2 information

**Expertise**	Multiferroic materials and interfaces, TEM and electron diffraction techniques
**Editor’s comments**	While this reviewer can appreciate the likely influence of your observations, they request additional data in order to quantitatively support your claim of a polarization gradient in the different samples you study.

Reviewer #2 comments

Section	Annotated Reviewer Comments
**Remarks to the Author: Overall significance**	The authors used many different techniques, e.g., DFT, S/TEM, PFM, XRD, etc., to characterize the PTO films grown on STO substrates. It is very interesting to see that the ‘single-domain’ PTO can be grown using a low-temperature solution epitaxy method. Also, it’s interesting to see that this system exhibits superior photovoltaic effect. The results are original and novel and should be of interest to others in this community. However, I have big concerns about two arguments claimed by the authors. On one hand, they should re-consider how to clarify the structural issue and the polarization gradient they claimed here. On the other hand, they should cite and credit more previous work, since without that, the claims can hardly be appropriately discussed in this work.<br><br>**For further consideration at *Nature Communications*, the authors**

	**should try and provide better support for the phase structure of PTO, as well as on the nature of the domains and domain wall structures, as requested below. They should perform additional experimental work (AED/4D-STEM, XRD, TOFSIMS) to support the observation of the claimed polarization gradient.**
**Remarks to the Author: Impact**	Yes, this paper will influence the way of synthesizing the functional ferroelectric and piezoelectric materials. Possibly their properties can be further enhanced due to implementation of this method.
**Remarks to the Author: Strength of the claims**	Below are my comments that the authors should consider to improve quality of the manuscript.<br><br>1. The authors claimed that they revealed a nontrivial polarization gradient throughout the films, which is up to ~500 nm?!. As one of the key findings, they argued that PTO is tetragonal phase, which is fully correct under normal conditions. But under the condition of ignorable interface mismatch strain, where the flexo- term is absent, the authors should instead seriously consider the rational of monoclinic PTO phase due to presence of such long-range in-plane polarization (Fig. 3c and Fig. S8). See App. Phys. Lett. 74, 2059 (1999); AIP Advances 6, 105208 (2016). On this regard, the authors should also consider to image oxygen atoms, e.g., using ABF or iDPC, to very the important phase structure of PTO.<br><br>2. Regarding the out-of-plane polarization gradient, the authors measured the polar Ti displacements at different places throughout the film using HAADF STEM. The images (Fig. S8) suggests that thickness and/or imaging conditions of the panel images are different. By doing simple simulations, the authors will immediately know that the polar displacement value is very sensitive to varied thickness under the same imaging conditions. This implies that the polarization gradient concluded this way is questionable or unreliable. For such a thick film, the polarization-strain relation usually holds. Some other measures, e.g., measuring distance dependent c/a ratio using SAED/4D-STEM across the film and XRD with grazing incident angle, should be helpful to provide additional evidence. See Nat. Commun. 12, 5322 (2021); Nat. Mater. 10, 963 (2011).<br><br>3. A polarization gradient along one direction actually means a metastable charged state of the ferroelectric. Somehow, this is similar to uncompensated polarization component along the direction normal to the plane of a Neel wall. From energy point of view, this is unstable and certain stabilization mechanism should be present. See Nat. Commun. 7, 12385 (2016). The authors should confirm whether the polarization gradient really exist. From Fig. 2g,2h, one can see crossed fine domain stripes. This possibly tells us that the PTO is not 'single domain', but with 90deg domains. Thus, the author should further clarify the phase, domain and domain wall structures, so as to better understand the large photovoltaic effect. See Nat. Nanotech. 5, 143 (2010); Nat. Commun. 5,

	4677 (2014); ACS ami 9, 6539 (2017).<br><br>4. Again, relating to the polarization gradient, the authors argued that a composition gradient could account for its presence. Analogously to Fig. S7i, the authors should provide solid evidence about the composition change as a function of distance using TOFSIMS. Which element is deficient/rich? What is the change of composition ratio as a function of distance? Meanwhile, the authors should explain why they choose 1.25:1 Pb:Ti ratio during film preparation?<br><br>5. In my view, the authors over-exaggerate the role of electronic polarization screening at the PTO/Nb-STO interface. Although high-Nb doping can provide better electron screening, the octahedral linking at the interface, which also participate in the screening, cannot be avoided. See Phys. Rev. B 98, 020102(R) (2018). For this reason, the authors should carefully evaluate the role of lattice mismatch, between BFO, KNO, PTO, PZT and STO, in solution epitaxy. Since usually when the lattice mismatch is large, multi-orientation domains may prevail in PTO. See Phys. Rev. B 67, 054107 (2003). On the other hand, should the authors consider different plane terminations, SrO or TiO2, to evaluate their screening capability?<br><br>6. There exists another controversial argument: Fig. S3f shows that the polarization direction is difficult to be switched. But in Fig. S11, they present the photovoltaic I-V data, that the polarization can be switched. So what is the real fact?<br><br>Overall, the arguments from the authors are not convincing, and more stringent data analysis and experiments should be carried out to clarify these puzzles.
**Remarks to the Author: Reproducibility**	Reproducibility should be no problem, given their growth of BFO, KNO and PZT on the Nb-STO substrates.

nature portfolio

GUIDED OPEN ACCESS

nature physics nature communications communications physics

Open research evaluation

General information

Guidelines for Transparency and Openness Promotion (TOP) in Journal Policies and Practices ("TOP Guidelines")

The recommendations and requests in the table below are aimed at bringing your manuscript in line with common community standards as exemplified by the TOP Guidelines. While every publisher and journal will implement these guidelines differently, the recommendations below are all consistent with the policies at Nature Portfolio. In most cases, these will align with TOP Guidelines Level 2.

FAIR Principles

The goal of the recommendations in the table below related to **data or code** availability is to promote the FAIR Guiding Principles for scientific data management and stewardship (*Scientific Data* **3**: 160018, 2016). The FAIR Principles are a set of guidelines for improving 4 important aspects of digital research objects: **F**indability, **A**ccessibility, **I**nteroperability and **R**eusability.

ORCID

ORCID is a non-profit organization that provides researchers with a unique digital identifier. These identifiers can be used by editors, funding agencies, publishers, and institutions to reliably identify individuals in the same way that ISBNs and DOIs identify books and articles. Thus the risk of confusing your identity with another researcher with the same name is eliminated. **The ORCID website** provides researchers with a page where your comprehensive research activity can be stored.

Springer Nature collaborates with the ORCID organization to ensure that your research contributions (as authors and peer reviewers) are correctly attributed to you. Learn more here.

Specific advice

Data availability
**Data Availability Statement**
Thank you for including a Data Availability Statement. In order to adhere to community standards for transparency and reproducibility, we encourage you to amend this so as to make it clear which data are available and under what conditions.<br><br>Please find more information about Data Availability Statements and Springer Nature's data policies here.
**Code availability and citation**

Thank you for including a Code Availability statement and for your willingness to make custom code available. However, we note that you have only indicated that custom code are available upon request. To adhere to community standards and promote transparency in research, we encourage the Code Availability Statement to indicate whether and how the code or algorithm can be accessed, including any restrictions to access. Public release of custom software may be required for publication in a Nature Portfolio journal.

Upon publication, Nature Portfolio journals consider it best practice to release custom computer code in a way that allows readers to repeat the published results. Code should be deposited in a DOI-minting repository such as Zenodo, Gigantum or Code Ocean and cited in the reference list following the guidelines described in our policy pages. Authors are encouraged to manage subsequent code versions and to use a license approved by the open source initiative. Full details about how the code can be accessed and any restrictions must be described in the Code Availability statement.

We also provide a Code and Software submission checklist that you may find useful.

Reporting & reproducibility

All source data underlying the graphs and charts presented in the main figures must be made available as Supplementary Data (in Excel or text format) or via a generalist repository (eg, Figshare or Dryad). This is mandatory for publication in a Nature Portfolio journal, but is also best practice for publication in any venue.

The following figures require associated source data: Fig. 3b and Fig. 4a-d.

Nature Portfolio journals allow unlimited space for Methods. The Methods must contain sufficient detail such that the work could be repeated. It is preferable that all key methods be included in the main manuscript, rather than in the Supplementary Information. Please avoid use of “as described previously” or similar, and instead detail the specific methods used with appropriate attribution.
