# Supplementary material for: Solution epitaxy of polarization-gradient ferroelectric oxide films with colossal photovoltaic current
Source: Nat Commun. 2023 Apr 24;14:2341. doi: 10.1038/s41467-023-37823-z (PMC10126087; doi:10.1038/s41467-023-37823-z)
Supplement: Supplementary file 2 — Supplementary Information [file 41467_2023_37823_MOESM2_ESM.pdf]

Supplementary Information

Solution epitaxy of polarization-gradient ferroelectric oxide films with colossal photovoltaic current

Chen Lin[1#], Zijun Zhang[2#], Zhenbang Dai[3#], Mengjiao Wu[1], Shi Liu[4], Jialu Chen[1], Chenqiang Hua[5], Yunhao Lu[1,5], Fei Zhang[6], Hongbo Lou[6], Hongliang Dong[6], Qiaoshi Zeng[6], Jing Ma[7], Xiaodong Pi[1,8], Dikui Zhou[1,9], Yongjun Wu[1,9], He Tian[2*], Andrew M. Rappe[3], Zhaohui Ren[1,9*], Gaorong Han[1*]

[1] State Key Laboratory of Silicon Materials, School of Materials Science and Engineering, Zhejiang University, Hangzhou, 310027, China.

[2] Center of Electron Microscope, School of Materials Science and Engineering, Zhejiang University, Hangzhou, 310027, China.

[3] Department of Chemistry, University of Pennsylvania, Philadelphia, Pennsylvania 19104-6323, USA.

[4] School of Science, Westlake University, Hangzhou, 310024, China.

[5] Zhejiang Province Key Laboratory of Quantum Technology and Device, Department of physics, Zhejiang University, Hangzhou, 310027, China.

[6] Center for High Pressure Science and Technology Advanced Research, Shanghai, 201203, China.

[7] State Key Lab of New Ceramics and Fine Processing, School of Materials Science and Engineering, Tsinghua University, Beijing, 100091, China.

[8] Institute of Advanced Semiconductors, Hangzhou Innovation Center, Zhejiang University, Hangzhou, 311215, China.

[9] Research Center for Intelligent Sensing, Zhejiang Lab, Hangzhou, 311100, China.

# These authors contributed equally to this work.

* Corresponding authors, e-mails: renzh@zju.edu.cn; hetian@zju.edu.cn; hgr@zju.edu.cn.

This Supplementary Information file includes:

Supplementary Figure 1–15

Supplementary Table 1–2

Supplementary References

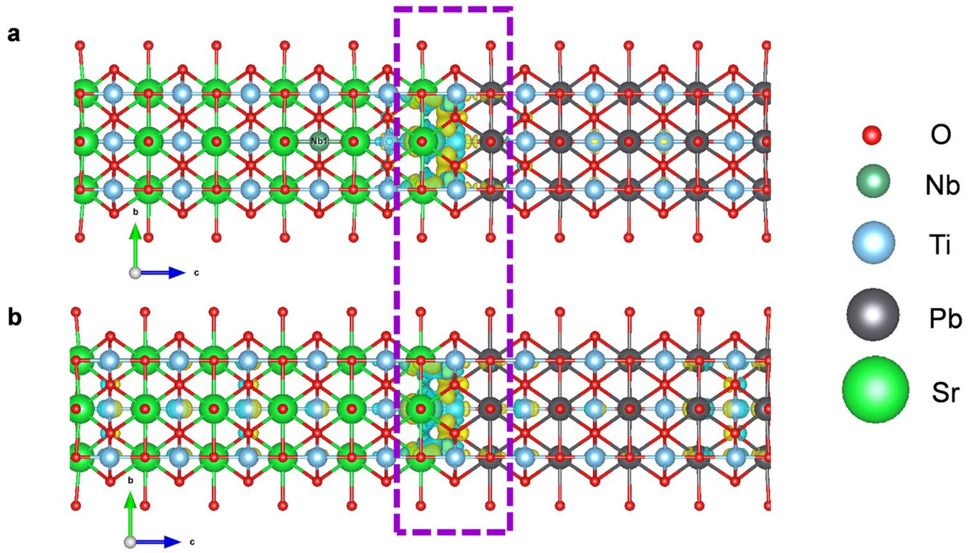

Supplementary Fig. 1 | Calculations of the interface formation energy. a, **b** Differential charge density of PTO/Nb:STO (**a**) and PTO/STO (**b**), where the dashed purple rectangle represents for the interfacial areas. Thermodynamic calculation results indicate that the formation energy of PTO/Nb:STO interface is 0.32 eV lower than that of PTO/STO interface. Both PTO/Nb:STO and PTO/STO systems exhibit charge transfer at the interface, but it is notably stronger in **a**. In addition, noticeable charge separation has been observed in both PTO and STO sides in **b**. These results demonstrate that ferroelectric polarization is fully screened at the PTO/Nb:STO interface while the screening in PTO/STO is realized not only by interfacial charge transfer but also by polarized STO and charge separation in PTO within several unit cells near the interface.

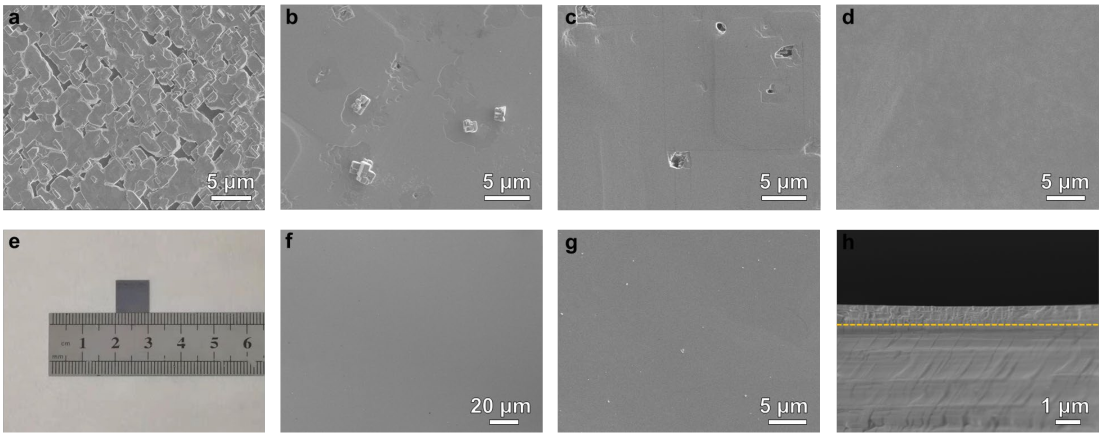

Supplementary Fig. 2 | The growth of PTO films on STO substrates with different Nb doping concentrations. a–d SEM images of PTO films on STO (**a**), 0.05 wt% Nb:STO (**b**), 0.5 wt% Nb:STO (**c**), 0.7 wt% Nb:STO (**d**). **e**, **f** Digital photo (**e**) and large-scale SEM image (**f**) of PTO film on 0.7 wt% Nb:STO substrate. PTO adopted a typical island growth on STO. In contract, continuous and smooth PTO films in a large-scale have been observed on Nb:STO substrate with 0.7 wt% Nb doping. **g**, **h** SEM images of the surface (**g**) and the cross-section (**h**) of PTO film on 1 wt% Nb:STO. The dashed line denotes the position of the interface.

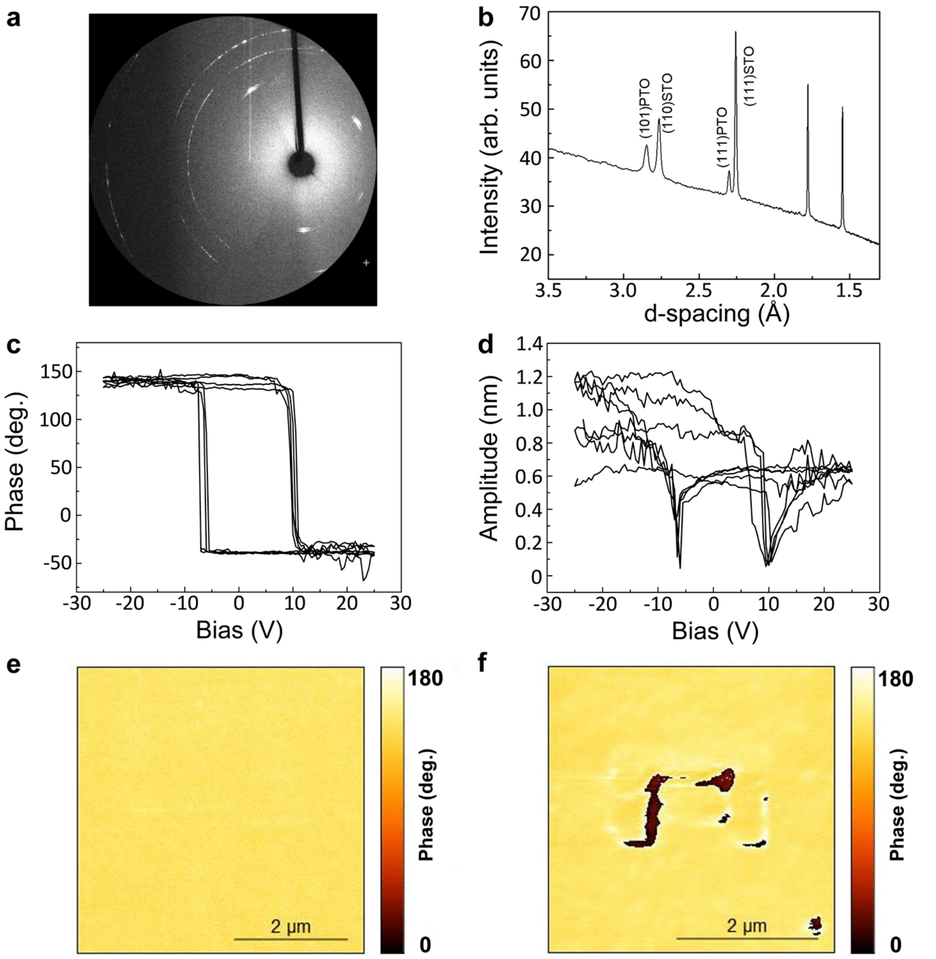

Supplementary Fig. 3 | PTO film with single-crystal and single-domain structure. **a**, **b** Two-dimensional synchrotron radiation XRD image (**a**) and integrated one dimensional synchrotron radiation XRD pattern (**b**) of PTO film on Nb:STO. The experimentally determined unit cell parameters of PTO are a=b=3.902 Å, c=4.152 Å, c/a=1.0641, α=β=γ=90° (The standard data of PTO are a=b=3.905 Å, c=4.156 Å, c/a=1.0643, α=β=γ=90°). The continuous powder rings on the two-dimensional diffraction image are background spurious diffraction signal from the upstream beamstop made of polycrystalline tungsten carbide. The sample signal is characterized as single-crystal spots in pairs. **c**, **d** Piezoresponse phase curve with a hysteresis loop (**c**) and amplitude curve with a “butterfly-shape” loop (**d**) of PTO film. **e**, **f** Out-of-plane PFM phase image (**e**) and PFM phase image recorded after writing box-in-box patterns with 25 V reverse dc bias using a biased conductive tip (**f**) of PTO film.

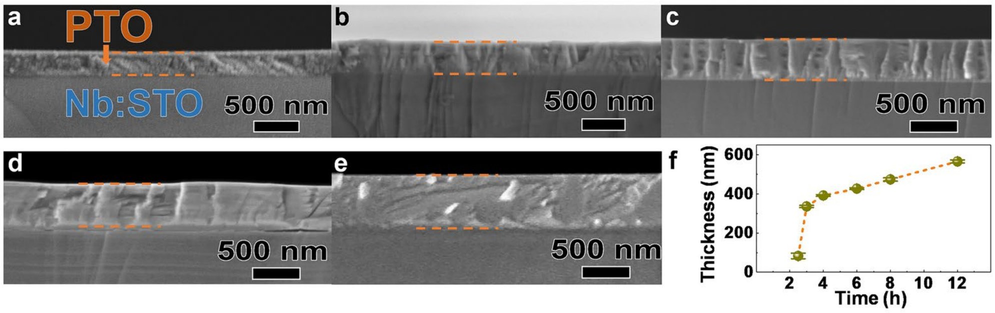

Supplementary Fig. 4 | A time-dependent thickness evolution of PTO film on Nb:STO substrate. a–e Cross-sectional SEM images of PTO/Nb:STO interface obtained by hydrothermal synthesis for 3 h (**a**), 4 h (**b**), 6 h (**c**), 8 h (**d**) and 12 h (**e**). **f** Experimentally extracted film thickness as a function of the hydrothermal reaction time. Each error bar denotes the s.d. of the film thickness of three samples hydrothermally synthesized at the same time.

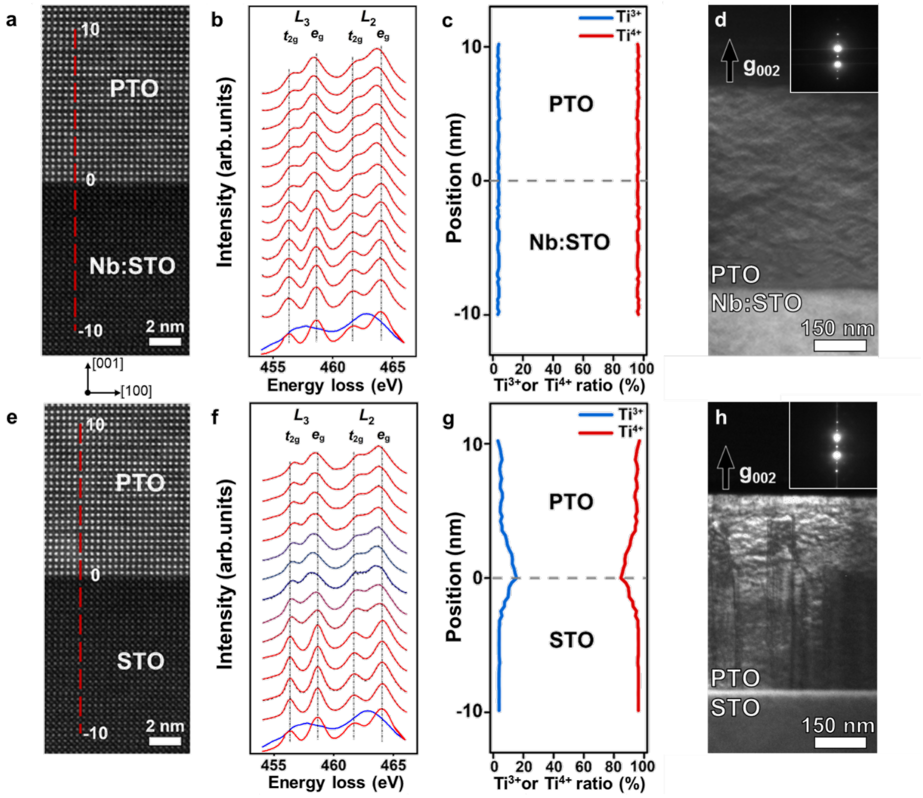

Supplementary Fig. 5 | Characterization of PTO/Nb:STO interface and PTO/STO interface. **a**, **e** Atomic-level cross-sectional HAADF-STEM images of PTO/Nb:STO interface (**a**) and PTO/STO interface (**e**), where position zero represents the interface. **b**, **f** A series of Ti-$L_{2,3}$ spectra across the interface from PTO to Nb:STO (**b**) and PTO to STO (**f**) plotted with black dots which were respectively acquired according to the red dashed line in **a** and **e**. A series of red solid lines in **b** and colored solid lines in **f** show the fit models of each experimental spectrum according to the reference spectra of Ti^{3+} (blue) and Ti^{4+} (red), both of which are shown at the bottom. **c**, **g** Ti^{3+} and Ti^{4+} content across the PTO/Nb:STO interface (**c**) and PTO/STO interface (**g**) obtained from fitting the spectra in each profile of **b** and **f** with normal Ti^{3+} and Ti^{4+}, where position zero represents the interface. **d**, **h** Cross-sectional dark-field TEM (DFTEM) images of PTO/Nb:STO (**d**) and PTO/STO (**h**), insets are the selected area electron diffraction (SAED) patterns. They were taken by the reflection of **g** = **002** to clarify the 180° domain structure. A domain with a polarization vector **P** gives rise to a bright contrast for $\mathbf{g} \cdot \mathbf{P} > 0$ [1]. Compared with PTO/Nb:STO, the inversion of contrast in PTO film indicates that 180° domains form near the PTO/STO interface.

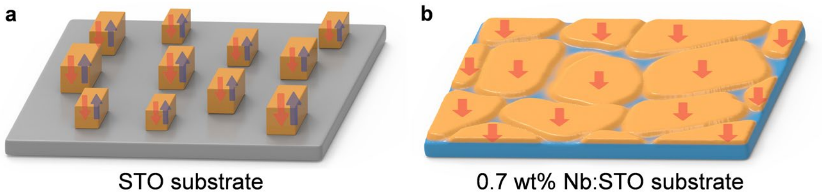

Supplementary Fig. 6 | The growth mode of PTO on STO substrate altered by electronic polarization screening at the interface. a Three-dimensional PTO crystals initially grow on undoped STO substrate, ferroelectric polarization of which is mainly stabilized by a formation of 180° domains (Supplementary Fig. 5h). **b** Two-dimensional PTO crystals initially grow on Nb:STO substrate, ferroelectric polarization of which is fully stablized by the electronic screening at the interface. Arrows in each PTO crystal denote their polarization direction. The wetting condition, which is crucial for a two-dimensional layer-by-layer-like growth, is achieved in PTO epitaxially growth on Nb:STO substrate due to the lower interface energy brought by electronic polarization screening.

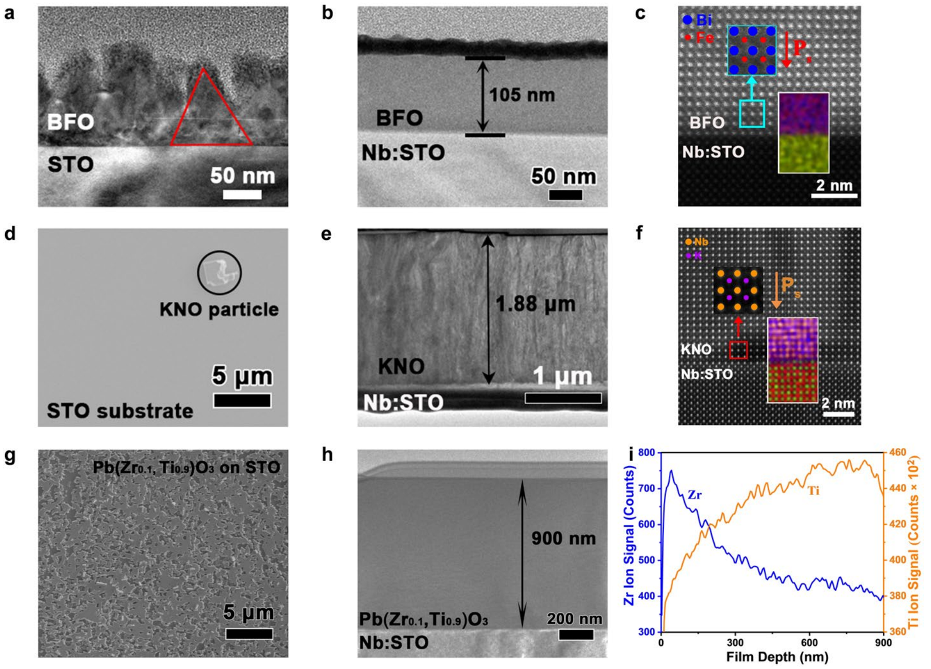

Supplementary Fig. 7 | The universality of the electronic polarization screening driven low-temperature solution epitaxy of ferroelectric oxide films. a Cross-sectional TEM image of BFO/STO, implying an island growth mode. **b** Cross-sectional TEM image of BFO/Nb:STO, showing a film thickness of ≈105 nm and a sharp interface. **c** Atomic-scale HAADF-STEM image of BFO/Nb:STO interface. Left inset is the enlarged image of the cyan rectangle, upward shift of Fe ions indicates the out-of-plane polarization of BFO is downward. Right inset shows the corresponding atomic-scale EDX map. Bi, Fe, Sr and Ti correspond to purple, red, green and cyan, respectively. **d** SEM image of KNO on STO substrate, showing that only a few KNO particles on STO substrate. **e** Cross-sectional TEM image of KNO/Nb:STO, showing a film thickness of ≈1.88 μm and a sharp interface. **f** Atomic-scale HAADF-STEM image of KNO/Nb:STO interface. Left inset is the enlarged image of the red rectangle, upward shift of K ions indicates a downward polarization of KNO. Right inset shows the corresponding atomic-scale EDX map. K, Nb, Sr and Ti correspond to purple, orange, red and green, respectively. **g** SEM image of $Pb(Zr_{0.1},Ti_{0.9})O_3$ on STO substrate, showing an island growth mode. **h** Cross-sectional TEM image of $Pb(Zr_{0.1},Ti_{0.9})O_3$/Nb:STO, showing a film thickness of ≈900 nm and a sharp interface. **i** Time-of-flight secondary-ion mass spectra (TOF-SIMS) for $Pb(Zr_{0.1},Ti_{0.9})O_3$ film.

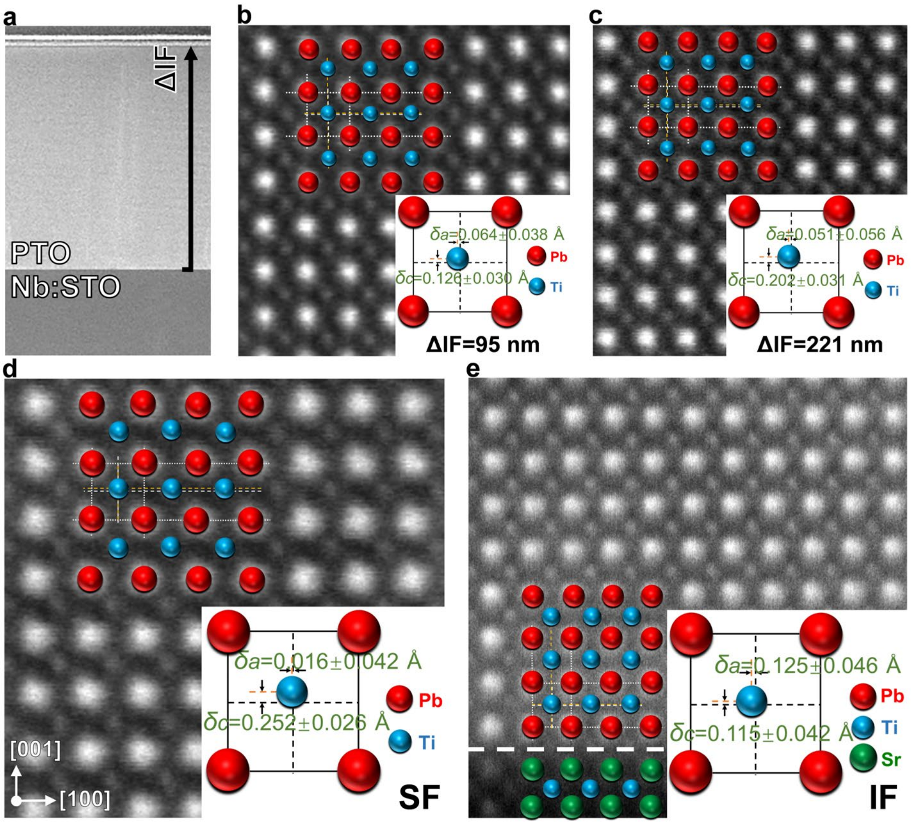

Supplementary Fig. 8 | Measurement of the polarization gradient throughout PTO film. a Cross-sectional TEM image of PTO/Nb:STO heterostructure. 'IF', 'SF', and 'ΔIF' represent the interface, the surface and the distance from the interface, respectively. **b**–**e** Atomic-scale cross-sectional HAADF-STEM images of regions with different ΔIF in PTO film: ΔIF ≈ 95 nm (**b**), ΔIF ≈ 221 nm (**c**), ΔIF ≈ 530 nm (**d**), ΔIF ≈ 0 nm (**e**). Insets are the schematic illustrations of the average-displacement measurement of Ti ions relative to the center of the four nearest Pb ion columns in each corresponding region. 'δ_a' and 'δ_c' denote the off-center displacements of the Ti ions along *a*-axis and *c*-axis, respectively.

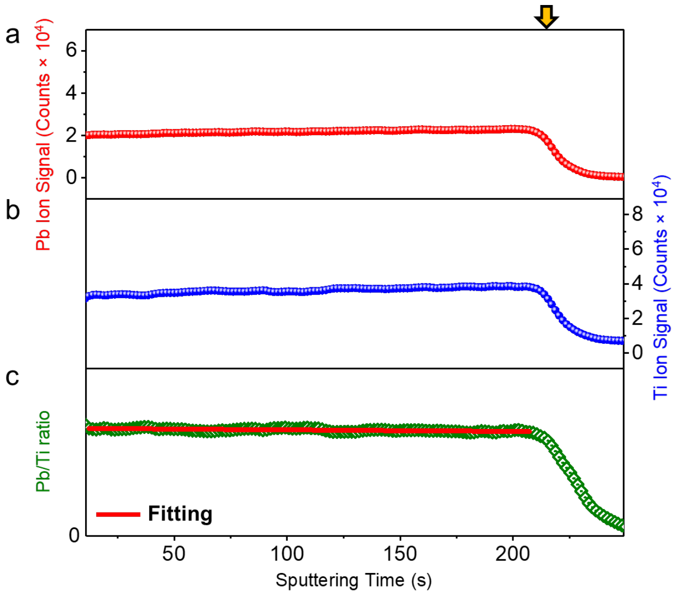

Supplementary Fig. 9 | TOF-SIMS for the depth profiling of PTO film. a Pb ion signal as a function of the sputtering time. **b** Ti ion signal as a function of the sputtering time. The sputtering time is generally linear with the film depth. Both signals are background corrected. **c** The ratio of Pb and Ti ion signals as a function of the sputtering time. The yellow arrow points to an abrupt change of the signals, corresponding to the interface of PTO/Nb:STO. The red solid line in **c** fits the linear relationship between the Pb/Ti ratio (in bulk of PTO film from the surface to the interface: the sputtering time before ~200 s) and the sputtering time.

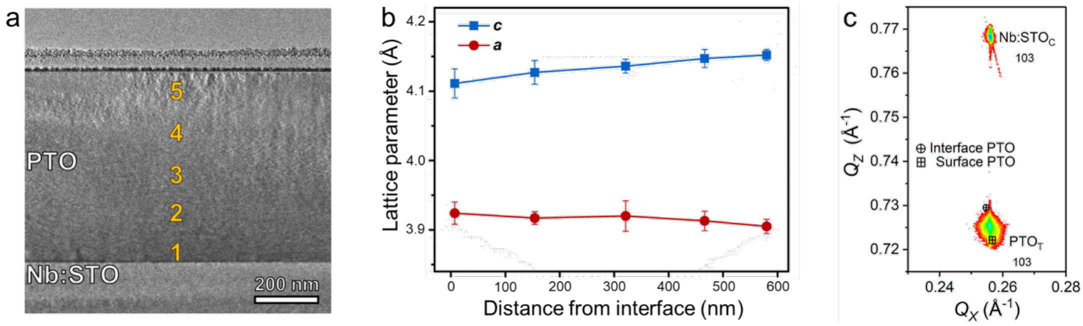

Supplementary Fig. 10 | Characterizations of structure gradient throughout PTO film. a Cross-sectional TEM image of PTO/Nb:STO. The areas for nanobeam electron diffraction (NBED) experiments are marked with numbers of 1-5. **b** Lattice parameter *a* and *c* as a function of the distance from PTO/Nb:STO interface determined from NBED patterns. **c** Off-axis X-ray RSM study about the pseudocubic 103-diffraction condition for PTO/Nb:STO. Diffraction peak positions according to the NBED analysis of the interface (corresponding to area 1 in **a**) and the surface (corresponding to area 5 in **a**) of PTO film are marked with circle and square, respectively.

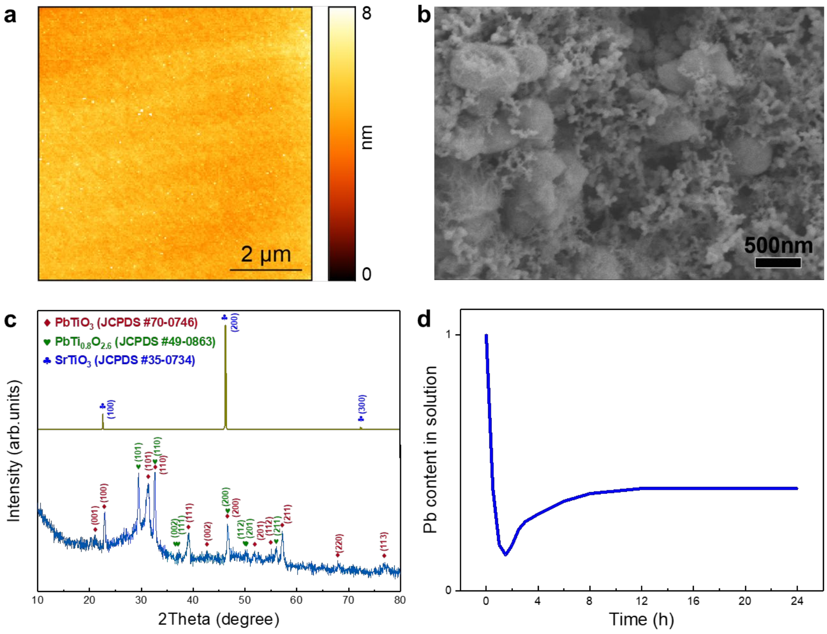

Supplementary Fig. 11 | Competitive growth between films and particles in hydrothermal system. a–c PTO/Nb:STO hydrothermally synthesized for 1 h. AFM topography image of Nb:STO surface (**a**) and SEM image of particles (**b**). Both of them were obtained in the same hydrothermal autoclave. XRD patterns of PTO/Nb:STO (yellow curve) and particles (blue curve) (**c**). **d** Schematic of Pb content in the hydrothermal solution as a function of the hydrothermal reaction time. Compared to the film hydrothermally synthesized after 12 h, PTO (00l) diffraction peaks cannot be actually observed in the sample hydrothermally synthesized for only 1 h. Meanwhile, $PbTiO_3$ (JCPDS #70-0746) and $PbTi_{0.8}O_{2.6}$ (JCPDS #49-0863) particles in the hydrothermal autoclave demonstrate a relatively good crystallinity, which confirms that the epitaxial growth of PTO film is highly limited during this stage due to the competitive consumption of reagents by the growth of particles.

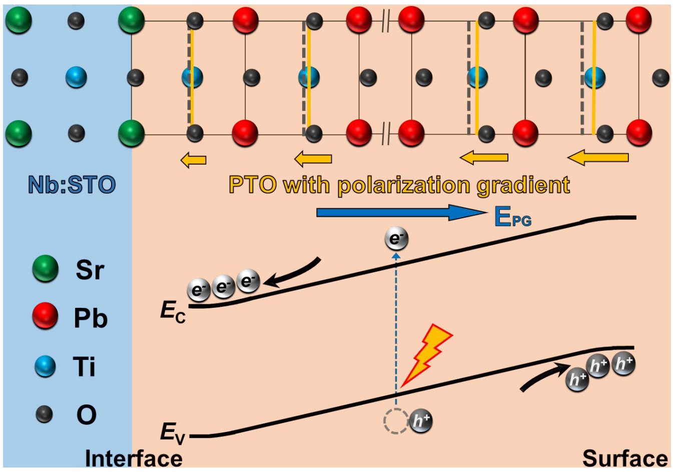

Supplementary Fig. 12 | Polarization gradient enhanced the photoresponsivity in PTO film. Schematic illustration of the built-in electric field $\mathbf{E}_{\mathbf{PG}}$ within the film induced by the polarization gradient (yellow arrows). This electric field is a driving force to separate photogenerated electrons and holes, and drives them to travel to the interface and the surface, respectively. Yellow solid lines and gray dashed lines respectively represent for the position of Ti ions and the mean position of two adjacent Pb-O layers along the polarization direction in each unit cell, 'E_C' and 'E_V' denote the energy at the bottom of conduction band and the top of valence band.

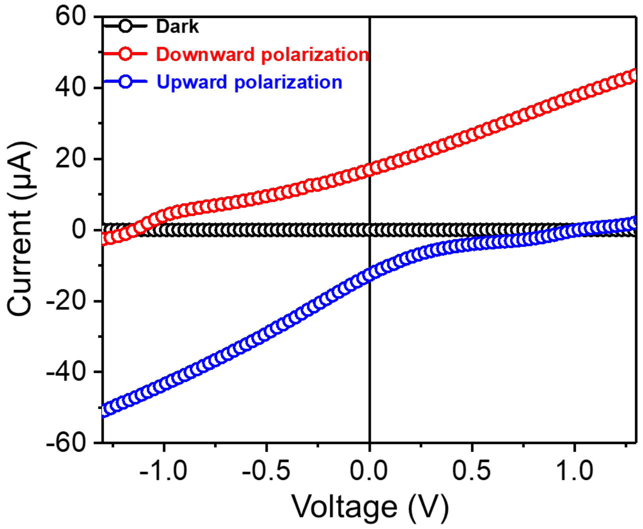

Supplementary Fig. 13 | *I*-*V* characteristics of PTO film with opposite ferroelectric polarization measured under illumination of I_{light} = 500 mW/cm^2 and dark conditions.

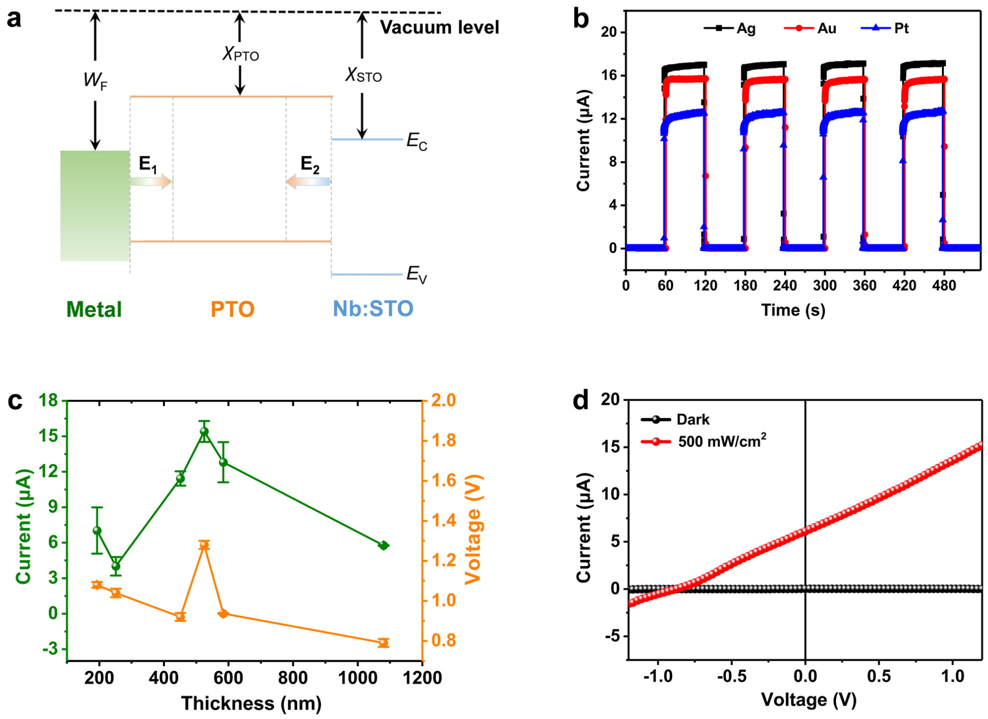

Supplementary Fig. 14 | The contribution of Schottky barriers to photovoltaic current at the surface and the interface. a Schematic of the energy band diagram of metal/PTO/Nb:STO structure, where $\mathbf{E_1}$ and $\mathbf{E_2}$ are electric fields originated from Schottky barriers between metal/PTO surface and PTO/Nb:STO interface, respectively. The magnitude of net built-in electric field $\mathbf{E_{net}}$ of two back-to-back Schottky barriers can be expressed as $|\mathbf{E_{net}}| = |\mathbf{E_2}| - |\mathbf{E_1}| \approx W_F - \chi_{STO}$, where $\chi_{STO} < W_F$ (χ_{STO} is the electron affinity energy of STO)[2]. **b** Short-circuit current response to the switching of light on and off at the zero bias, when I_{light} of 500 mW/cm^2 illuminated PTO films with different top electrodes. A metal electrode with a larger W_F would lead to a larger $|\mathbf{E_{net}}|$, thus resulting in a larger photovoltaic current. The opposite results in **b** demonstrate that Schottky barrier makes few contribution to the photovoltaic current in our work, where the W_F of Ag, Au and Pt are 4.26 eV, 5.20 eV and 5.65 eV, respectively[3]. **c** Short-circuit current and open-circuit voltage under illumination of I_{light} = 500 mW/cm^2 as a function of PTO film thickness. Each error bar denotes the s.d. of the data measured on three samples with similar film thickness. **d** *I*-*V* characteristics of PTO film with a thickness of 1.08 μm under illumination of I_{light} = 500 mW/cm^2 and dark conditions.

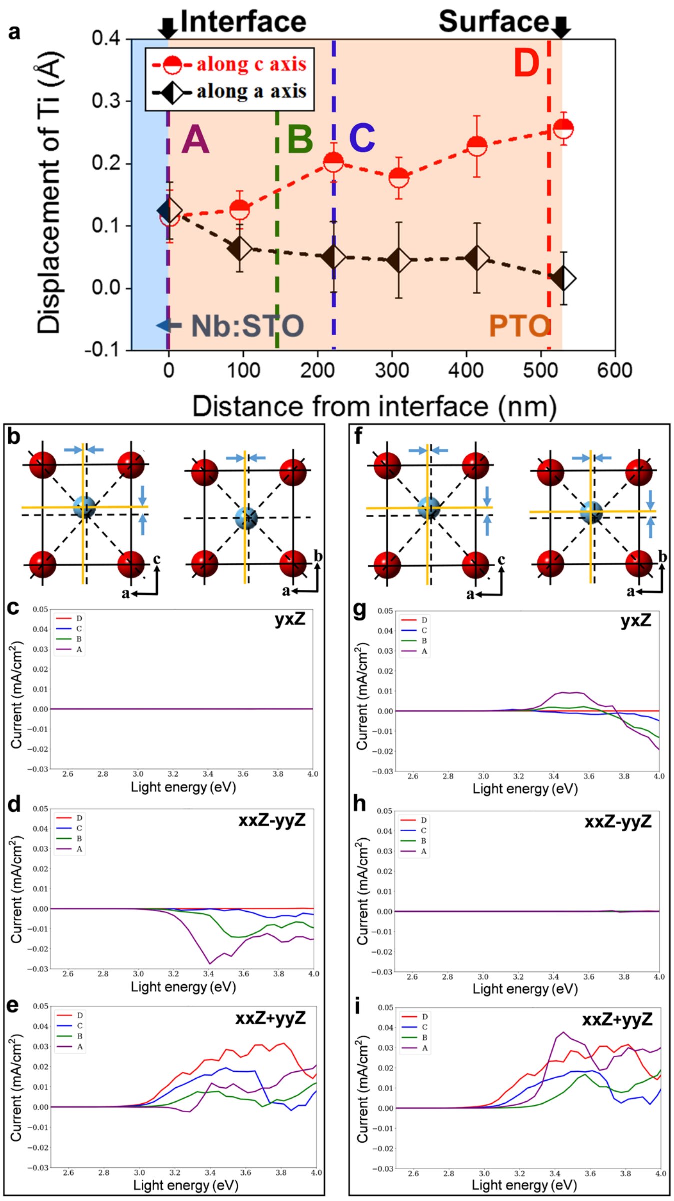

Supplementary Fig. 15 | Shift current simulations of gradient ferroelectrics. a Off-center displacements of the Ti ions as a function of the distance from the interface. The dashed lines marked A–D represent the parameters chosen for shift current calculations from first principles. When considering the displacements of Ti ions along *b*-axis, their magnitudes are assumed to be equal to that along *a*-axis. **b**, **f** Side view and top view of the displacements of Ti ions along *c*- and *a*-axis (**b**), or along *c*-, and *a*-, *b*-axis (**f**). **c**–**e**, **g**–**i** The first-principle shift currents for PTO with chosen four parameters marked A–D, resulting different components of shift current tensors for the displacements of Ti ions along *c*- and *a*-axis (**c-e**): *yxZ* (**c**), *xxZ-yyZ* (**d**), *xxZ+yyZ* (**e**), or along *c*-, and *a*-, *b*-axis (**g-i**): *yxZ* (**g**), *xxZ-yyZ* (**h**), *xxZ+yyZ* (**i**).

By allowing the Ti ions to move along *ab*-plane, the symmetry of a normal PTO is broken so that either *xxZ* and *yyZ* components are no long equal (Ti move along *a*- and *c*-axis) or the *yzZ* component can be non-zero (Ti move along *a*-, *b*-, and *c*-axis). Therefore, our calculation suggested that the shift current constitutes to the experimental photocurrent with less than 1/25 contribution**.**

$$J_c(\psi) \propto [\cos 2\psi \ \ \sin 2\psi] \begin{bmatrix} \sigma_{xxZ} & \sigma_{xyZ} \\ \sigma_{yxZ} & \sigma_{yyZ} \end{bmatrix} \begin{bmatrix} \cos 2\psi \\ \cos 2\psi \end{bmatrix}$$

$$= \sigma_{xxZ} \cos^2 2\psi + \sigma_{xyZ} \cos 2\psi \sin 2\psi + \sigma_{yxZ} \cos 2\psi \sin 2\psi + \sigma_{yyZ} \sin^2 2\psi$$

$$= \sigma_{yxZ} \sin 4\psi + \frac{(\sigma_{xxZ} - \sigma_{yyZ})}{2} \cos 4\psi + \frac{(\sigma_{xxZ} + \sigma_{yyZ})}{2} \qquad (1)$$

ψ is the rotation angle of the polarization direction of the incident light.

Supplementary Table 1 | Electric properties of STO substrate and Nb:STO substrates

Nb Doping concentration (wt%)	0	0.1	0.5	0.7	1
**Resistivity (Ω·m)**	$1–10^6$	0.08	0.05	0.007	0.0035
**Conductivity (S/m)**	-	12.5	20	142.86	285.71
**Mobility (cm^2/Vs)**	-	6.5	8.5	8.5	9.0
**Electron concentration (cm^{-3})**	$\sim 10^{14}$	1.20×10^{19}	1.47×10^{19}	1.05×10^{20}	1.98×10^{20}

The data of electric properties of 0.1–1 wt% Nb:STO were provided by the manufacturer (Shenyang Baijujie Corporation). Although data of 0.1 wt% Nb:STO substrates were given, 0.05 wt% Nb:STO substrates were used instead in our work. They are confirmed to have similar electric properties. The electron concentration of STO was obtained from ref.[4], in which we take the thermal excitation into consideration due to a hydrothermal temperature of 200°C, i.e. 473K.

Supplementary Table 2 | Summary of the reported photovoltaic properties in the bulk phase of ferroelectrics

Materials(type)-Publication year	J_{SC} / μA/cm^2	V_{OC} / V	Light wavelength / nm	I_{light} / mW/cm^2	Photorespon-sivity (J_{SC} /I_{light}) / A/W
**$PLZT_{(3/52/48)}$ (Ceramic film)[5] -2004**	1.7	0.8	near-ultraviolet	150	**1.13×10^{-5}**
**$BiFeO_3$ (Bulk single crystal)[6] -2009**	7.35	≈0.08	532	<20	**≈3.675×10^{-4}**
**$BiFeO_3$ (Epitaxial film)[7] -2010**	≈0.28	0.3	435	0.75	**≈3.73×10^{-4}**
**$BiFeO_3$ (Bulk single crystal)[8] -2011**	1	13	405	8000	**1.25×10^{-7}**
**$[KNbO_3]_{0.9}[BaNi_{1/2}Nb_{1/2}O_{3-\delta}]_{0.1}$ (Ceramics)[9] -2013**	0.1	3.5	700-900	4	**2.5×10^{-4}**
**$BaTiO_3$ (Epitaxial film)[10] -2014**	7	0.65	360	750	**9.33×10^{-6}**
**$[PbTiO_3]_{0.65}[BiNi_{2/3}Nb_{1/3}O_{3-\delta}]_{0.35}$ (Ceramics)[11] -2015**	0.116	10	400-780	200	**5.8×10^{-7}**
**Mn-doped $BiFeO_3$ (Epitaxial film)[12] -2017**	15	3.1	515	2500	**6.0×10^{-6}**
**La-substituted $BiFeO_3$ (Epitaxial film)[13] -2018**	≈2.5	≈0.55	halogen lamp	100	**2.5×10^{-5}**
**$BiFeO_3$ (Epitaxial film)[14] -2020**	28.8	0.715	1 Sun AM 1.5G	100	**2.88×10^{-4}**
**Fe:$KTa_{0.41}Nb_{0.59}O_3$ (Bulk single crystal)[15] -2020**	≈0.206	6	405	≈1111.11	**≈1.85×10^{-7}**
**(iso-pentylammonium)$_2$(ethylammonium)$_2Pb_3I_{10}$ (Bulk single crystal)[16] -2021**	1.5	0.8	637	127	**1.18×10^{-5}**
**This work：PTO (Epitaxial film)**	2153	1.15	375	500	**4.306×10^{-3}**

Supplementary References

1. Aoyagi, K. et al. Diffraction contrast analysis of 90° and 180° ferroelectric domain structures of $PbTiO_3$ thin films. *Sci. Technol. Adv. Mater.* **12**, 034403 (2011).
2. Sawa, A. et al. Fermi level shift in $La_{1-x}Sr_xMO_3$ (M=Mn, Fe, Co and Ni) probed by Schottky-like heteroepitaxial junctions with $SrTi_{0.99}Nb_{0.01}O_3$. *Appl. Phys. Lett.* **90**, 252102 (2007).
3. Kim, B., Choi, S. H., Zhu, X. Y. & Frisbie, C. D. Molecular tunnel junctions based on π-conjugated oligoacene thiols and dithiols between Ag, Au, and Pt contacts: effect of surface linking group and metal work function. *J. Am. Chem. Soc.* **133**, 19864–19877 (2011).
4. Crawford, J. C. Ferroelectric field effect studies at low temperatures. *Ferroelectrics* **1**, 23–30 (1970).
5. Ichiki, M. et al. Photovoltaic effect of lead lanthanum zirconate titanate in a layered film structure design. *Appl. Phys. Lett.* **84**, 395–397 (2004).
6. Choi, T., Lee, S., Choi, Y. J., Kiryukhin, V. & Cheong, S. W. Switchable ferroelectric diode and photovoltaic effect in $BiFeO_3$. *Science* **324**, 63–66 (2009).
7. Ji, W., Yao, K. & Liang, Y. C. Bulk photovoltaic effect at visible wavelength in epitaxial ferroelectric $BiFeO_3$ thin films. *Adv. Mater.* **22**, 1763–1766 (2010).
8. Alexe, M. & Hesse, D. Tip-enhanced photovoltaic effects in bismuth ferrite. *Nat. Commun.* **2**, 256 (2011).
9. Grinberg, I. et al. Perovskite oxides for visible-light-absorbing ferroelectric and photovoltaic materials. *Nature* **503**, 509–512 (2013).
10. Zenkevich, A. et al. Giant bulk photovoltaic effect in thin ferroelectric $BaTiO_3$ films. *Phys. Rev. B* **90**, 161409 (2014).
11. Liu, H. et al. Large photovoltage and controllable photovoltaic effect in $PbTiO_3$-$Bi(Ni_{2/3+x}Nb_{1/3-x})O_{3-\delta}$ ferroelectrics. *Adv. Electron. Mater.* **1**, 1400051 (2015).
12. Matsuo, H., Noguchi, Y. & Miyayama, M. Gap-state engineering of visible-

light-active ferroelectrics for photovoltaic applications. *Nat. Commun.* **8**, 207 (2017).

13. You, L. et al. Enhancing ferroelectric photovoltaic effect by polar order engineering. *Sci. Adv.* **4**, eaat3438 (2018).
14. Wang, M. X. et al. Polarization-enhanced bulk photovoltaic effect of $BiFeO_3$ epitaxial film under standard solar illumination. *Phys. Lett. A* **384**, 126831 (2020).
15. Huang, F. et al. Photovoltaic properties in an orthorhombic Fe doped KTN single crystal. *Opt. Express* **28**, 34754–34760 (2020).
16. Han, S. G. et al. Tailoring of a visible-light-absorbing biaxial ferroelectric towards broadband self-driven photodetection. *Nat. Commun.* **12**, 284 (2021).
